# Information management for high content live cell imaging

**DOI:** 10.1186/1471-2105-10-226

**Published:** 2009-07-21

**Authors:** Daniel Jameson, David A Turner, John Ankers, Stephnie Kennedy, Sheila Ryan, Neil Swainston, Tony Griffiths, David G Spiller, Stephen G Oliver, Michael RH White, Douglas B Kell, Norman W Paton

**Affiliations:** 1 Manchester Centre for Integrative Systems Biology, School of Chemistry, and Manchester Interdisciplinary Biocentre, University of Manchester, 131, Princess St, Manchester, M1 7DN, UK; 2 Centre for Cell Imaging, School of Biological Sciences, Bioscience Research Building, Crown St., Liverpool, L69 7ZB, UK; 3 School of Computer Science, Kilburn Building, The University of Manchester, Oxford Road, Manchester, M13 9PL, UK; 4 Department of Biochemistry, University of Cambridge, Sanger Building, 80 Tennis Court Road, Cambridge, CB2 1GA, UK

## Abstract

**Background:**

High content live cell imaging experiments are able to track the cellular localisation of labelled proteins in multiple live cells over a time course. Experiments using high content live cell imaging will generate multiple large datasets that are often stored in an ad-hoc manner. This hinders identification of previously gathered data that may be relevant to current analyses. Whilst solutions exist for managing image data, they are primarily concerned with storage and retrieval of the images themselves and not the data derived from the images. There is therefore a requirement for an information management solution that facilitates the indexing of experimental metadata and results of high content live cell imaging experiments.

**Results:**

We have designed and implemented a data model and information management solution for the data gathered through high content live cell imaging experiments. Many of the experiments to be stored measure the translocation of fluorescently labelled proteins from cytoplasm to nucleus in individual cells. The functionality of this database has been enhanced by the addition of an algorithm that automatically annotates results of these experiments with the timings of translocations and periods of any oscillatory translocations as they are uploaded to the repository. Testing has shown the algorithm to perform well with a variety of previously unseen data.

**Conclusion:**

Our repository is a fully functional example of how high throughput imaging data may be effectively indexed and managed to address the requirements of end users. By implementing the automated analysis of experimental results, we have provided a clear impetus for individuals to ensure that their data forms part of that which is stored in the repository. Although focused on imaging, the solution provided is sufficiently generic to be applied to other functional proteomics and genomics experiments. The software is available from:

## Background

### Introduction

Recent advances in biological experimental techniques have often been geared towards increasing throughput of data. In microscopy, the combination of fluorescently labelled proteins, combined with automated focus and image capture, has led to a large increase in "high content screening" of cells, for phenotype and protein localisation in response to a variety of environmental perturbations (e.g. [1-4]). The majority of these assays deal with cells at single time points, but more challenging, and potentially more useful for a systems biology approach involving mathematical modelling, is being able to track cellular functions in a number of individual live cells over a period of time.

**Figure 1:**
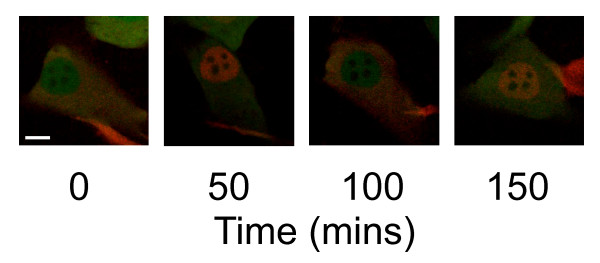
**Time course observing movement of fluorescently labelled protein involved in the NF-κB signalling system**. Scale bar represents 10 μm. SK-N-AS cells transfected with p53dsredXP and EGFP.

We have been using high content cell imaging techniques to explore the NF-κB signalling system. NF-κB proteins are a family of transcription factors involved in the regulation of cell division, apoptosis and inflammation [5,6]. NF-κB is released from inhibitor kappa-B (IκB) in the cytosol and translocates into the nucleus where it activates transcription of target genes, including that of its inhibitor IκB. IκB binds to NF-κB and transports the active complex back into a dormant cytoplasmic localisation [5,7]. The relationship between NF-κB and IκB leads to delayed negative feedback, which generates oscillatory behaviour in NF-κB localisation [5]. These oscillations can be studied effectively using fluorescently tagged NF-κB and IκB proteins (Figure 1). However, the pathway is complex, and high throughput screening of samples is proving to be essential for investigation of the mechanisms that regulate these dynamic processes [5]. Additionally, an important part of our work concerns the construction of an accurate dynamic mathematical model of the NF-κB pathway which must be fitted and verified using experimental data.

Traditionally, experimental details and results are recorded in lab books. This enables an experimentalist to quickly return to an experiment that they have performed; providing they can remember which book they recorded the information in. Whilst this may be sufficient for small self-contained experiments in small research groups, it does not, even at that scale, provide any facility to correlate seemingly disparate results and experiments between experimentalists unless links are drawn through discussions or by diligent project management. As the number of experiments performed and the number of measurements taken in each experiment increase, it becomes less likely that relevant associations will be identified, and more likely that important links will be overlooked, or at worst, experiments will be needlessly repeated.

Imaging experiments, such as those described, present additional problems, in that they generate large primary data sets with associated results that cannot be written into lab books. These may be stored on computer hard drives or DVDs, where typically only the experimentalist understands the precise system of nomenclature that they have used for their generated files, and experience suggests that even they may have some difficulty in discovering and interpreting datasets produced over an extended period.

In our specific environment, there is a requirement to archive in the region of 120 experiments per week, each one of which may be analysing up to 20 individual locations containing multiple cells over variable time courses. The image data are stored on RAID arrays providing a reasonable level of integrity for the data, but the amount of data generated presents a number of significant challenges for both information management and data analysis.

Systematic analysis of the time-series data gathered has been facilitated through the use of CellTracker [8,9], which allows fluorescent intensity in the cytoplasm and nucleus of individual cells to be quantified and recorded automatically. This is performed by allowing the experimentalist to define nuclear and cytoplasmic boundaries for cells of interest, and then using a particle filter algorithm to track those boundaries as they move within the captured image field from frame to frame. The software measures the fluorescent intensities within each boundary for each captured frame, and then calculates the ratio between them.

However, the requirement for effective information storage and indexing has necessitated the design and implementation of an experimental metadata repository. A repository for high throughput imaging data in the context described above:

1. Enables associations to be identified between experimental details, results and subsequent analyses.

2. Avoids repetition of experiments, as previous work is recorded.

3. Supports identification of relevant experiments; these may be from the same microscope, similar experimental conditions or have produced similar results.

### Requirements & Contribution

To address the problem outlined and provide the benefits described, requirements for the information management system were identified through an iterative series of meetings with both experimentalists and modellers. Initially these comprised of examining how data were manually gathered, recorded, and used, followed by discussions of what desirable attributes an information management system for these data would possess. The requirements identified were:

R1. Representation of experimental metadata and associated results.

R2. Efficient mechanisms for data capture.

R3. Efficient search mechanisms to identify experiments from metadata.

R4. Automated annotation of data derived from the images to allow identification of experiments whose results met certain criteria.

This paper presents an information management solution for high throughput cell imaging experiments, illustrated in the context of the NF-κB pathway, that addresses the above requirements by:

R1. Developing a new model that can capture both experiment descriptions and associated results, specifically descriptions of experimentalists, materials, protocols and microscope settings, as well as the output from the CellTracker software.

R2. Deploying the model using the Pedro data capture tool [10] to capture data that is then stored in a native XML database. The data capture tool utilises specially written plugins that allow the automatic import of microscope settings from microscope output files, as well as the re-use of previously described protocols and materials by importing them directly from the database.

R3. Providing a web-based search interface that uses canned (pre-defined) queries to retrieve details and results of experiments of interest. This provides the means to search over both experimental metadata and results.

R4. Computing summaries of the key features of time series image data (the change in fluorescence intensity of different cellular compartments over time and oscillations between these compartments) using a specifically developed algorithm, which are stored using the database from (R2) and accessed by way of the interface of (R3).

### Related Work

Our requirements centre on the effective management of experimental metadata and results. Such solutions exist for local storage of other types of functional genomic experiments (e.g. maxd [11] for genomic expression data) and a variety of repositories cater for public storage and dissemination of experimental results (e.g. ArrayExpress [12], PRIDE [13]). Here we discuss available infrastructures for the management of microscopy image data, and note that at present no available solution provides for the local management of experimental metadata and results from high content imaging experiments.

Individual microscope manufacturers produce file formats specific to their own hardware and software (e.g. [14]). The associated software infrastructures typically do not provide complex search facilities across multiple experiments, and metadata associated with images is generally limited to details of imaging settings, omitting experimental context. In general the emphasis in vendor software is on experimental analysis rather than archiving and retrieval.

The Carl Zeiss LSM (Laser Scanning Microscope) format is a good example of a vendor microscope output. It is generated by Carl Zeiss confocal microscopes and the resulting files may be viewed with the LSM Image Browser [14]. The format consists of two parts, a Microsoft Access format MDB (Microsoft Access Database) file which describes microscope settings and references the image file for each location observed, and a series of .lsm (Laser Scanning Microscope) files that contain the imaging data for the observed locations. This records much of the data relevant to an imaging experiment, but does not record information relating to sample preparation and experimental design. Additionally, as there is a single image database for each experiment conducted, it is not straightforward to search across multiple experiments.

The Open Microscopy Environment (OME) is a fully featured repository for image data and associated metadata [15] designed to be utilised as a local archive. It is able to read image data from a wide variety of microscope formats, and is becoming ever more widely used. Once images are imported into the database, the user may annotate them using either pre-defined or custom tags. Additionally, external modules may be deployed that can interface with and use the data stored in OME, for example FindSpots [16]. OME is dependant upon the Bio-Formats library [17] for reading and writing to different microscopy image formats, and as such is limited in the metadata it can extract directly from the image files. For example, it is unable to extract the microscope settings from Zeiss LSM files. OME's emphasis is, however, on the management of images from microscopy experiments, rather than on the use of high throughput imaging as a functional genomics technique, as in this paper.

The Cell Centred Database (CCDB) is an online repository for managing and sharing image data [18]. Whilst originally geared to storing electron micrographs [19], it has been extended to encompass a wide range of microscopy techniques. All submitted data must be accompanied by project, experimental and protocol metadata. Along with original image data, CCDB stores analysis products derived from these, such as segmentations into substructures and reconstructions into three-dimensional objects. Submission to CCDB and subsequent annotation of images and input of instrument parameters is performed manually through a series of web-forms. CCDB is a solution for presenting and disseminating analysed datasets to a wider community, however recently it has started to be evolved to support work within an experimental environment, with the introduction of MyCCDB [18]. MyCCDB provides a personalised login allowing individuals or groups to upload data and assign privileges for others to view and manipulate the data without it being published on the public site.

The Centre for Bio-Image Informatics at Santa Barbara have produced Bisque (Bio-Image Semantic Query User Environment) [20]. It is centred around the storage of biological images and associated metadata. Personalised logins may be registered and individuals may upload images that may then be kept private or made public. Uploaded images may be directly annotated with drawing objects or else via associated meta-data tags. Images may also be analysed with a number of tools specifically tailored for analysis of retinal tissues (e.g. segmentation of layers, counting of nuclei) and microtubules (e.g. dynamics analysis and segmentation). Results of certain analyses are stored directly in the database, but there is no facility to search across them.

Over the last 15 years there has been a considerable amount of work on Content Based Image Retrieval (CBIR). CBIR aims to identify images or sequences of images based on the visual content of the images. This content is reduced to a set of features – perhaps colour, textures or shapes – that may then be compared to the features extracted from other images either by measuring some "distance" between them or applying some sort of probabilistic model to suggest how relevant an image may be [21]. The precise nature of the features extracted depends on the problem being addressed by the CBIR system.

CBIR has been applied experimentally to many fields of medical and biological imaging, e.g. asymmetry in brain MRI scans [22], features in X-rays [23], RNAi induced phenotypes of cells [24]. However, each system generated is optimized for a particular task and there are no generalised products available that are able to perform automatic feature extraction on the type of data that we gather other than CellTracker which we currently use.

CellTracker's feature extraction could be considered the first step in a CBIR system, however there is no requirement to identify specific images based on these extracted features, just a requirement to identify similar sets of extracted features. If a need were to be identified for identification of particular result sets based on aspects cellular morphology or another aspect of the captured images that are not explicitly extracted by CellTracker then some further form of CBIR may be an appropriate addition to our requirements list.

MyCCDB, OME and Bisque are strongly focused on image storage and retrieval, and address many of the problems which are faced by those who are generating large quantities of image data from disparate sources and those who need to make their image data available to a wider audience. Whilst our needs overlap with some of the features provided by these solutions, we differ in that there is no requirement to manage image data itself, but there is a specific requirement to record and manage the details of the context in which the images were acquired, and subsequently the numerical data derived from CellTracker analyses performed on them.

## Implementation

### Data Requirements

**Figure 2:**
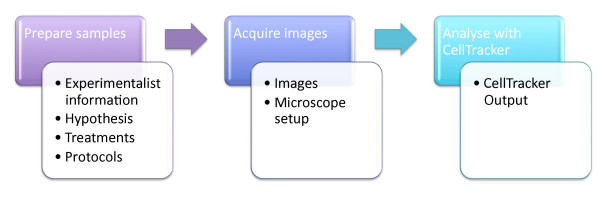
**Workflow diagram illustrating the process of conducting a microscopy experiment**. White boxes indicate the outputs from each stage that are required to describe the experiment.

In addressing R1 it is important to understand the nature of the experiments undertaken, the data generated and the requirements for access. Figure 2 is a workflow diagram describes a generic microscopy experiment and data that is output from or required to describe each stage.

The requirement for the model is that it must represent the output from experiments and results along with descriptive metadata. The metadata should, in keeping with guidelines devised for other experimental metadata [25,26], provide enough detail to validate the experimental methods used, minimise unnecessary repetition of experiments, and provide enough detail to repeat the experiment. There is no explicit requirement for the images to be archived along with the metadata, and as such it is reasonable to maintain a reference to the location of the image data, rather than storing the raw data in the database.

### The Model

**Figure 3:**
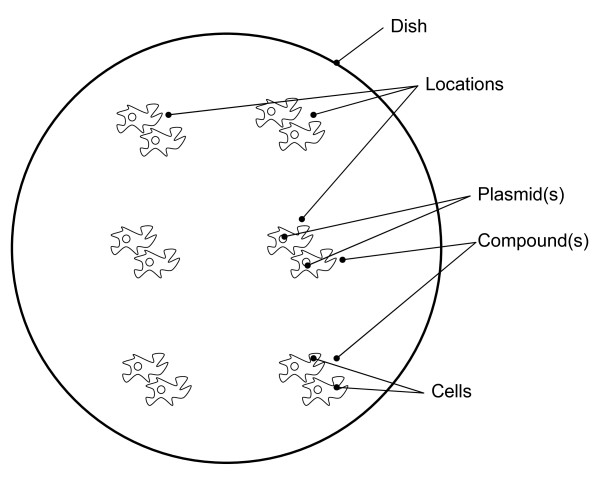
**Elements that form an experimental dish unit**.

The microscopy experiments conform to the set-up illustrated in Figure 3. Cells are observed in multiple locations on a dish. Cells may be transfected with one or more plasmids and treated with one or more compounds. The plasmids and compounds may be identical at every location across the dish or, in the case of high throughput screenings, may vary by location. Each experiment performed may have more than one dish. Associated with the dish, or locations on the dish, are additional treatments and potential environmental perturbations. These in turn have protocols associated with them. This arrangement of entities forms the core of a model that can be used to describe an experiment and its associated results. Beyond that, on a more abstract level, is a description of the context within which it was created.

**Figure 4:**
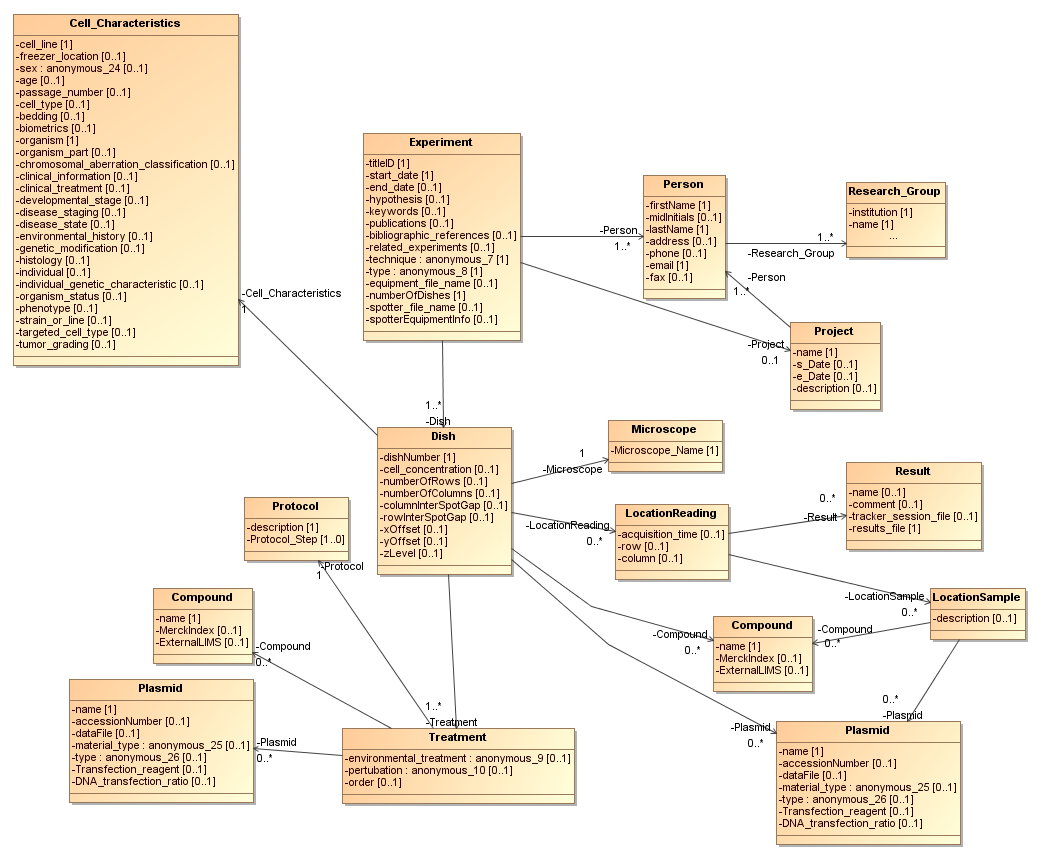
**UML Diagram illustrating relationships between metadata elements and experimentally generated elements**.

Figure 4 shows a UML diagram describing the relationships between the main objects that are associated with the *Experiment *class. Experiments are performed by people who belong to research groups. Experiments have a hypothesis, may be of various types (e.g., FISH (Fluorescence In Situ Hybridisation), FRET (Förster Resonance Energy Transfer) or Fluorescence), and may be performed using various techniques (e.g., confocal or wide field microscopy). Experiments can have many *Dish*es that were observed with a *Microscope*, as described above. The Experiment may be a spotted experiment, in which case cells treated in different ways may be applied to particular locations on the dish. Images are produced of *Location*s on the *Dish*, which once analysed, yield results that are directly related to that *Location*.

**Figure 5:**
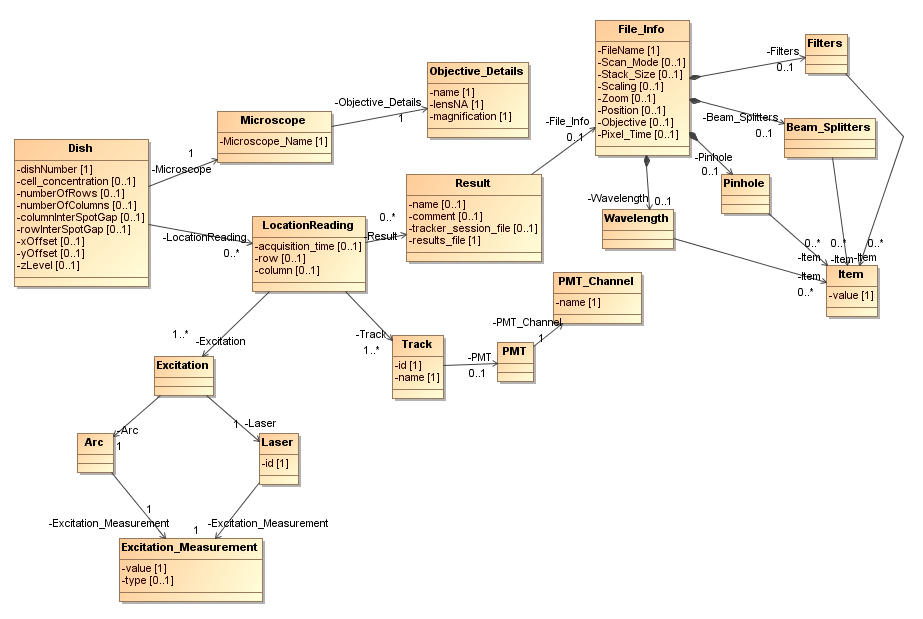
**Classes derived from Microscope data files**.

The model represents the physical and conceptual relationships between the elements that go to make up the experiment, but it must also capture the additional information provided by the microscope data files. The microscope records a large amount of information about its settings alongside the images it captures, such as laser intensities, filters, objectives, time points and tracks. This information is useful for validating procedures and replicating experimental configurations. Figure 5 shows the elements of the model that directly relate to the information provided in the *Microscope *data file and their relationship to the *Dish*, *LocationReading *and *Results *objects.

**Figure 6:**
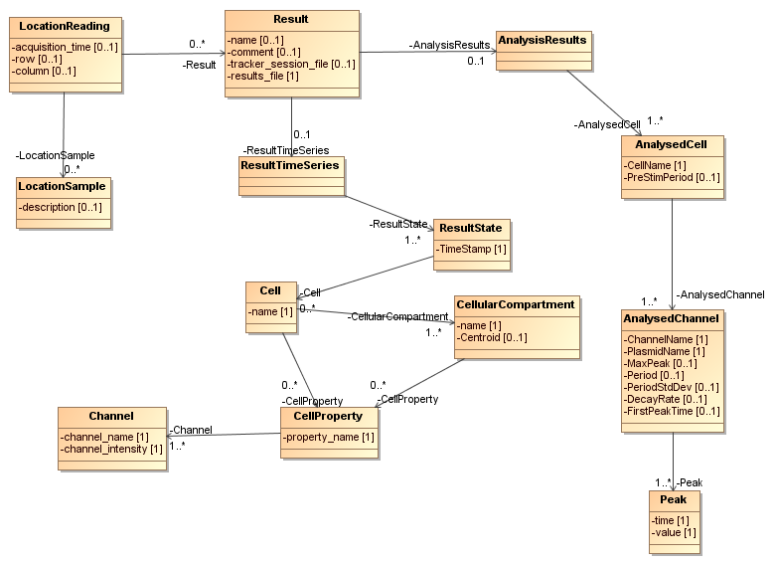
**How the Result data structure relates to Location Reading**.

The remainder of the model is populated from CellTracker output and subsequent analysis performed on this. As CellTracker outputs XML (see additional files 1, 2, 3, 4, 5 and 6), the model represents the CellTracker output format where a *ResultTimeSeries *has a sequence of *ResultState*s. CellTracker records the fluorescence in the nucleus and cytoplasm (*CellularCompartment*s) of each *Cell*. Both *Cell*s and *CellularCompartment*s have fluorescence data captured about them (*CellProperty*) on one or more *Channel*s. These *Channel*s have a *name *that can be related back to the wavelength of light (and hence tagged protein of interest) which was being recorded on that channel, and *intensity *of the fluorescence recorded. Additionally, we use this results section of the model to hold information derived from an automated analysis of the CellTracker output (described in "Summarisation of Results" below). For each recorded *Result *for a location on a dish, we generate *AnalysisResult*s. The *AnalysisResults *consist of one or more *AnalysedCell*s for that dish location, and for each of these we generate *AnalysedChannel*s corresponding to the *Channel*s that were recorded for that particular cell. The *AnalysedChannel*s contain the details (*Time*, ratio *value*) of any *Peak*s relating to the movement of fluorescence between cytoplasm and nucleus. The content of this section of the model is shown in Figure 6.

### Implementation of the Model

The data model is implemented as an XML Schema Definition (XSD), and thus the associated data are captured as XML. This is advantageous for two reasons. Firstly, XML is a de-facto standard for the transfer of biological data. Secondly, we are able to make use of existing software infrastructure for capturing, managing and accessing XML data. The full XSD, along with example data files, are available in the additional files.

### Data Capture

**Figure 7:**
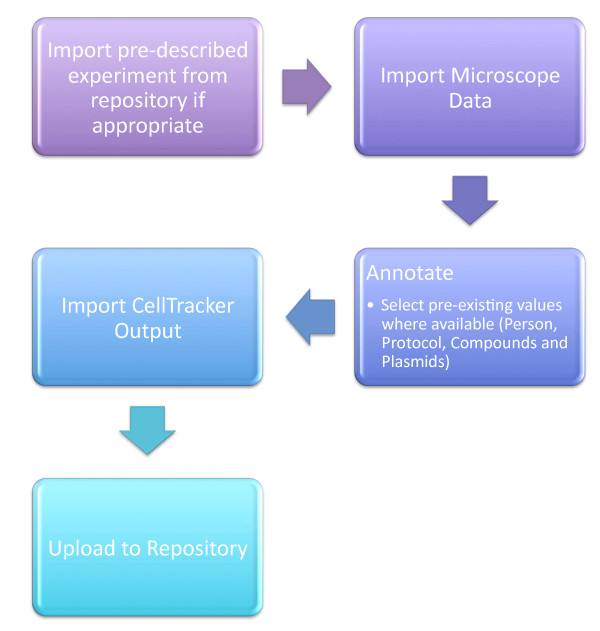
**Workflow diagram illustrating the process of annotating and uploading an experiment to the database using Pedro**.

The data capture workflow is illustrated in Figure 7. The data are captured using Pedro [27]. Pedro is a flexible model-driven data capture tool that is used to populate XML documents that adhere to a predefined schema. The use of Pedro as a data capture tool for cell imaging data is discussed elsewhere [10]. To fulfil R2, data capture for the repository must capture the information specified by the model but minimise the amount of form-filling which must be performed by the experimentalist. This is achieved in two ways:

1. Making use of smaller repositories that store model fragments relating to commonly used items, such as Researchers, Protocols, Plasmids and Compounds. These can be selectively added to the main document being edited in Pedro.

2. Extracting metadata and experimental structure from the microscope generated data files and the CellTracker output files.

The extraction of metadata from the microscope data initially populates a *Dish *document element with the correct number of *LocationReading *elements. These in turn are automatically annotated with the correct image data file names and microscope settings.

For each *LocationReading *an analysis file is produced by CellTracker, which populates the result elements with the relevant time series data. After capturing the data, it may be saved as an XML document, or directly submitted to the database.

Once stored documents may be imported directly from the database back into Pedro for further editing and updating – results may be added or removed,

### Data Storage

The Tamino [28] or eXist [29] native XML DBMS can be used to implement the repository, which allows us to directly store the XML documents generated by Pedro during data capture. Additionally, as discussed below, the use of native XML storage provides for convenient generation of web pages using XSLT (XSL Transformation).

### Data Access

Requirement 3 is for efficient searching over the archived metadata. Data needs to be accessed for two reasons, either updating, or searching and viewing. For updating, the data may be directly loaded back into Pedro from the database. For searching and browsing we have produced a web-accessible front end.

The front end is implemented as a series of Java Server Pages that send XQuery queries to the database and then transform the returned XML into HTML using XSLT documents.

After discussions with the experimentalists and modellers, initially ascertaining how data were currently consumed and subsequently examining what other questions may be asked of the data once stored in a database, the following requirements for querying the data were identified:

• List experiments by specific Experimentalist

• List experiments using a specific cell line

• List experiments performed on a specific date

• List experiments performed using a specific cell line and a specific plasmid

• List experiments performed using a specific compound treatment

• List experiments performed using a specific compound and specific plasmid

• List experiments performed using two specific plasmids

As such, the emphasis of the search interface is on finding specific experiments rather than on more complex tasks such as comparison of experiments. As a result, the database is performing the role of an experimental catalogue.

Additionally, requirements were identified for retrieval of experiments, the results of which have certain characteristics:

• Show results where an oscillation of a specific period (give or take a certain amount) has taken place.

• Show results where a change in whole cell luminescence has taken place at a specific rate (give or take a certain amount).

All of these queries have been implemented, but as new requirements are identified, it is generally straightforward to add new queries.

**Figure 8:**
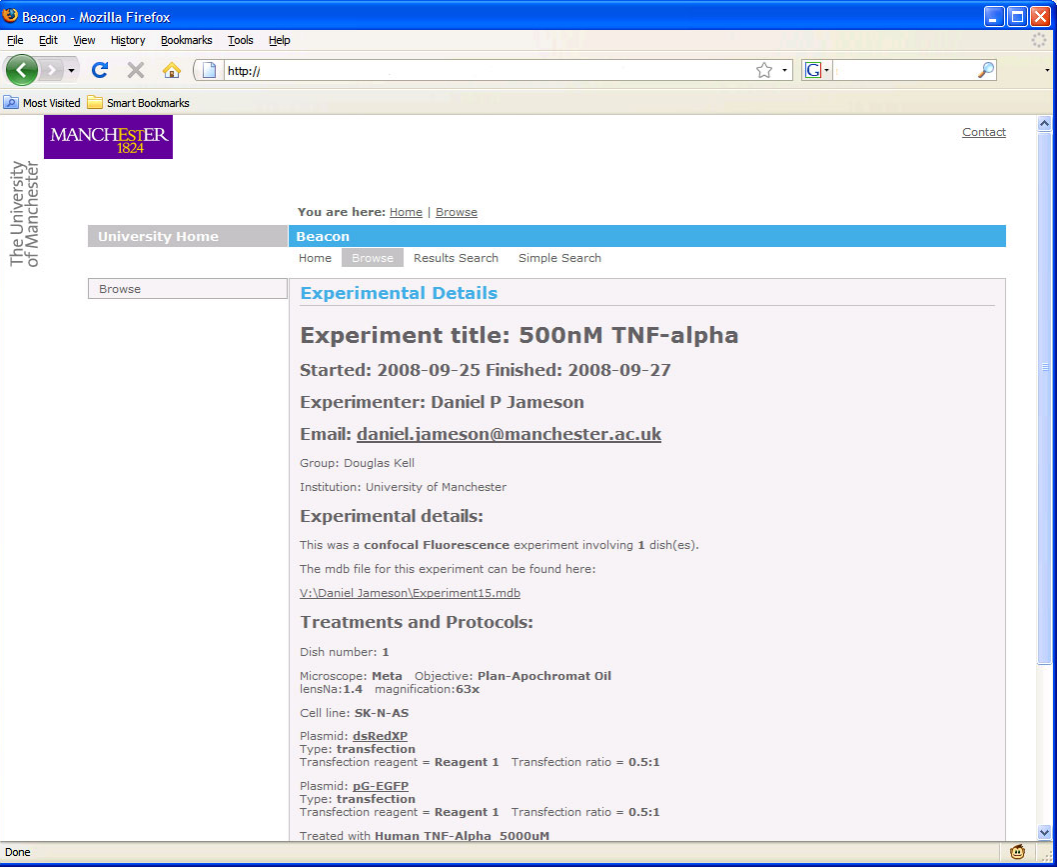
**Experimental details from the metadata stored in the database**. Canned search queries are in the list on the left hand side of the screen.

The results of queries that return experimental lists are represented as a table of experiment titles. Clicking through provides a summary of the experiment and protocols used (Figure 8). From there it is possible to click through to the results of that experiment.

Queries interrogating the results yield a table of experiment titles, and selecting one takes the user directly to the results, highlighting any locations that fulfilled the query request. In order to allow querying over the experimental results we have implemented a summarisation algorithm that is run when CellTracker analyses are imported into Pedro.

### Summarisation of Results

The facility to search the database for experiments whose results fit certain parameters, specified as R4, is important for modellers and experimentalists alike. We identified the following questions as being relevant for searching over experimental results:

Q1. Is there a movement of a measured fluorescent protein between the cytoplasm and nucleus, and if so when does this occur?

Q2. Are there subsequent movements resulting in an oscillation, and if so what is its period?

Q3. Is there a general trend in the overall level of measured fluorescence in a cell over time?

To meet these requirements, summaries are generated from the results of CellTracker analyses, which are stored in the database.

### Algorithm

**Figure 9:**
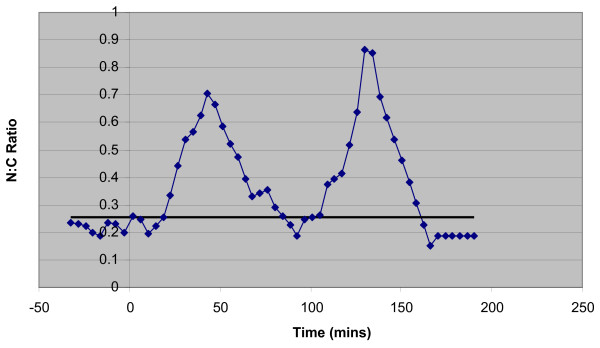
**A peak in Nuclear:Cytoplasmic ratio of labelled protein, denoting a translocation between cellular compartments**. *Also indicated are calculated detection thresholds and data points extrapolated by the peak detection algorithm*.

CellTracker generates a series of nuclear and cytoplasmic fluorescence intensities over time. By calculating and plotting the ratio between these intensities the translocation of a labelled protein from cytoplasm to nucleus may be observed as a peak (Figure 9). An algorithm has been developed to automate the detection of these peaks in the CellTracker output. This enables us to annotate the data as it is imported into the database with the times of Nuclear-Cytoplasmic translocations and the period of any oscillation, which in turn allows us to answer questions Q1 and Q2. Q3 is addressed by calculating a regression line through the whole cell fluorescence over time.

The peak detection algorithm takes as its input nuclear and cytoplasmic fluorescence intensity values over time. It returns peaks identified by time and nuclear:cytoplasmic (N:C) ratio and the period of any identifiable oscillation. The algorithm is implemented in Java and based upon the Tom O'Haver's PeakFinder function for MatLab [30]. This was chosen as it had been specifically designed to identify positive peaks in noisy time-series data, and provided several parameters that could be adjusted to fit the data gathered from cell imaging.

**Figure 10:**
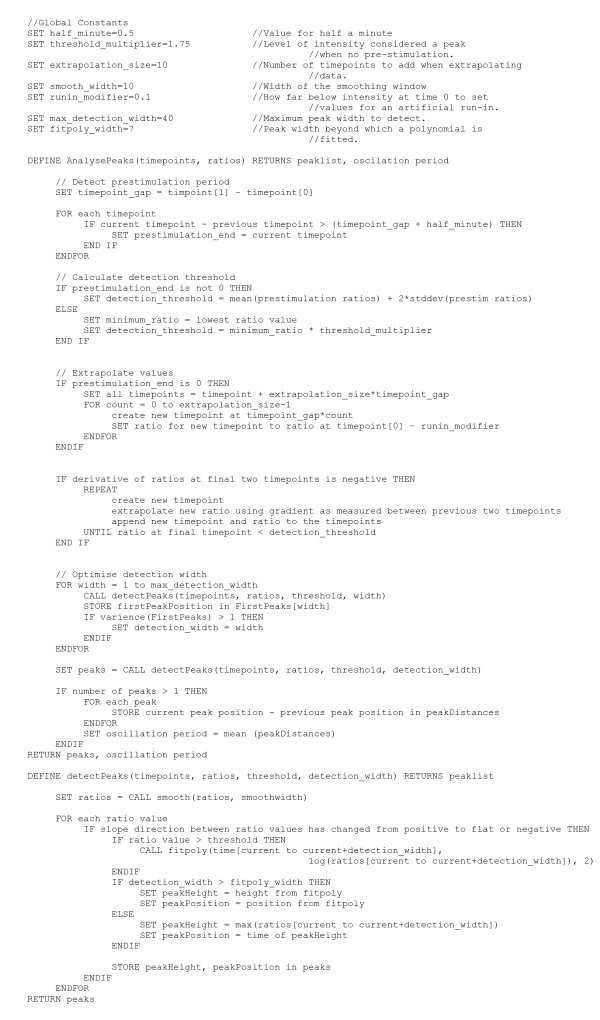
**Pseudocode for the peak detection algorithm**. fitpoly is a Java implementation of the MatLab Polyfit function which finds the coefficients of a polynomial which fits the specified data – fitpoly(x,y,n) where x and y are vectors of the x and y values and n is the order of the polynomial to be fitted.

The function accepts the data to be analysed along with parameters specifying the width of peaks to spot, a height threshold they must pass beyond (in our case this is a nuclear:cytoplasmic ratio), the width of the window to be used in the sliding average smoothing applied to the data, and a threshold gradient for the slope of the peak. The pseudocode for the algorithm is shown in Figure 10.

The algorithm has the following stages:

*1. Detect prestimulation*. Some experiments start when a chemical stimulus is added to the cells, others are run with a pre-stimulation period providing a base level for the N:C ratio. Adding the stimulus takes at least 30 seconds and hence an increase in the spacing of timepoints by at least this amount is indicative of a prestimulation period having been undertaken.

*2. Calculate detection threshold*. If a prestimulation has been undertaken, the detection threshold should be twice the standard deviation of the N:C ratios in the prestimulation period (the criteria used by the experimentalists). Otherwise set the threshold to be 1.75 times the minimum recorded N:C ratio – there is some tolerance with this value, however setting it much lower (1.5) or higher (2) increases the false positive and negative rates respectively.

*3. Extrapolate values*. If no prestimulation has occurred, the peak detection function is unlikely to identify the first peak, so a run-in of 10 timepoints with the N:C ratio at 0.1 below the starting value is prepended to the data. The peak detection function is also unlikely to a final peak that does not finish below the calculated detection threshold, hence if the final ratio values in the time series are a downward slope, the slope is extrapolated until it falls below the detection threshold. This is illustrated in Figure 7.

**Figure 11:**
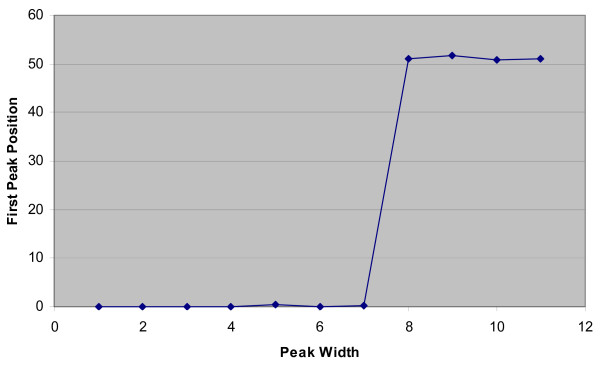
**Detected position of first peak with increasing peak detection width**.

*4. Optimise detection width*. The detection width (that is the width of peak, in numbers of time points, to be detected) to be used is determined by repeatedly calling the peak detection function with increasing detection widths. When the optimal detection width is encountered, there is a jump in the detected location of the first peak (Figure 11).

*5. Detection of peaks*. Derivatives of the data are smoothed using an average sliding window of 10 time points in width (Lower values introduced extra false positives within available data, higher values increased false negatives). If a maxima in the data is encountered and the ratio value is above the detection threshold then a peak is recorded. If the detection width is less than 7 time points (as implemented in the original algorithm [30]), the location and height of that local maximum is recorded. For larger detection widths, a second order polynomial is fitted to the data and the location and height of its maximum is recorded.

## Results

### Algorithm evaluation

Data sets from two experimenters comprising noisy and smooth data (a subjective observation of how easily real peaks are visually identified, generally a product of the protein being observed and the amount of stimulation applied to the cell) from three different cell lines were used to parameterise the algorithm (Table 1, also see additional files 7 and 8). Experiments involved inducing the NF-κB pathway with TNF-α and then observing the localisation of tagged proteins involved in that pathway. Three groups of unseen data were made available for testing the algorithm, which involved different experimenters and observed proteins (Table 2, also see additional files 7 and 9). The experimental protocol may be found in additional file 7.

**Table 1:** Training data used.

**Data Set**	**Experimenter**	**Cell Line**	**Data type**	**Pre-stimulation for baseline?**	**Cell Count**	**Tracks**
T1	1	SK-N-AS	Noisy	Yes	16	16

T2	1	SK-N-AS	Noisy	Yes	26	26

T3	1	SK-N-AS	Smooth	Yes	22	22

T4	1	SK-N-AS	Smooth	Yes	26	26

T5	2	SK-N-AS	Intermediate	No	25	32

T6	2	HeLa	Intermediate	No	51	97

T7	2	MEF	Intermediate	No	26	41

Where Tracks is larger than Number of Cells, multiple tagged proteins were being monitored in each cell.

**Table 2:** Test data used.

**Data Set**	**Experimenter**	**Cell Line**	**Data type**	**Pre-stimulation for baseline?**	**Cell Count**	**Tracks**
1	1	SK-N-AS	Intermediate	Yes	21	21

2	1	SK-N-AS	Smooth	Yes	21	21

3	1	SK-N-AS	Noisy	Yes	23	23

4	3	HeLa	Noisy	No	10	20

5	3	HeLa	Noisy	No	12	24

6	3	SK-N-AS	Noisy	No	12	24

7	3	SK-N-AS	Noisy	No	12	24

8	4	HeLa	Intermediate	No	102	102

### Method of evaluation

The algorithm analyses Nuclear:Cytoplasmic ratios from individual cells. As any subsequent analysis of protein movement is based upon the accurate detection of peaks in this data, the presence and absence of peaks in automatically and manually annotated results form a suitable metric by which to measure the effectiveness of the algorithm.

For each cell analysed a list of peaks detected by the algorithm is generated. This list comprises of the peak location (Time) and peak height (N:C ratio). These are then scored against manually observed peaks in the same data. The possible outcomes are:

• True positive (TP): the peak is detected by the algorithm and also observed as significant.

• False positive (FP): the peak is detected by the algorithm but not observed as significant.

• False negative (FN): no peak is detected by the algorithm but one is observed as significant.

Observations are subjective, in that they are performed by individuals who may have a preconceived idea of what their results should look like and also dependant on the particular parameters of an experiment. For example, depending on the treatment applied to them, some cell lines generally do not show any oscillations of the protein being observed between cytoplasm and nucleus, and hence an observer may be less likely to identify a small secondary peak as real.

The expected results from the test data sets were known in advance; the algorithm results for the test data were scored by the experimentalists.

### Results

The effectiveness of the algorithm is illustrated by Recall (TP/(TP+FN)) and Precision (TP/(TP+FP)). TP, FP and FN are True Positives, False Positives and False Negatives respectively. A precision score of 1.00 would mean that every peak identified was a true peak, but would not give an indication of how many true peaks were missed. A recall score of 1.00 would mean that all the peaks present in the data were correctly identified as peaks, but would not give an indication of how many non-peaks were identified as peaks. Taken together, these metrics give a good indication of the effectiveness of the algorithm. The training results (Table 3 and additional file 10) show an overall precision of 0.84 and a recall of 0.93. Results from test data, representative of the data that is stored in the database, are shown in Table 4 (also see additional file 10). Here the overall precision is shown to be 0.71 and recall is 0.83.

**Table 3:** Training data algorithm effectiveness.

**Data Set**	**Recall (TPR)**	**Precision**
T1	1.00	0.58

T2	0.89	0.84

T3	0.93	0.76

T4	0.94	0.97

T5	1.00	0.58

T6	0.89	0.93

T7	0.94	0.86

**Overall:**	**0.93**	**0.84**

TPR is True Positive Rate – TP/(TP+FN). Precision is TP/(TP+FP).

**Table 4:** Test data, algorithm effectiveness.

**Data Set**	**Recall (TPR)**	**Precision**
1	0.80	0.85

2	0.85	0.90

3	0.84	0.89

4	0.73	0.29

5	0.77	0.40

6	0.44	0.17

7	1.00	0.54

8	0.87	0.87

**Overall**	**0.83**	**0.71**

### Discussion of Algorithm Performance

With the training data, the algorithm performs well, successfully identifying over 90% of peaks in the data presented to it with a precision of 0.84. These were the data used to optimise the parameters of the algorithm and our approach to handling the data; as such, we expect good performance. This result also shows that our optimization has not been so strict as to yield a 100% score on the training data. Such a situation would be undesirable, indicating that the algorithm had little capacity to generalize beyond the training data. The true indicator of performance is through the analysis of unseen test data. With the test data the performance is less robust (identifying 83% of peaks with a precision of 0.71). However, almost half of the test data (45%) were rated as noisy by the experimentalists as opposed to 16% in the training data.

**Figure 12:**
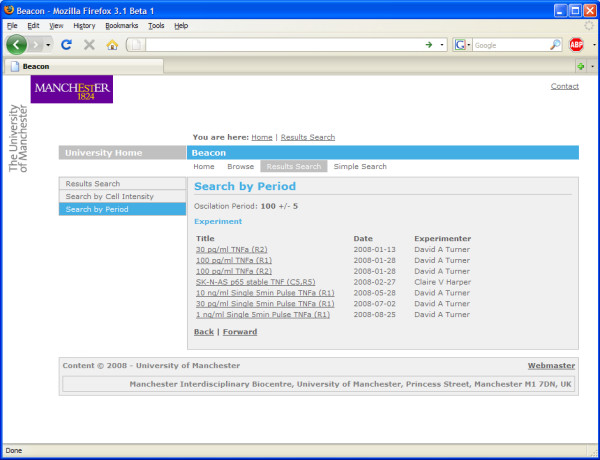
**Searching stored experiments using a specific period of oscillation**.

The automatically generated data summaries aim to facilitate the identification of experiments in the database that may have yielded results of interest. Although we do not successfully identify every peak in the data, given that experiments invariably measure multiple cells in multiple locations, we can expect that a search over the results (of the nature Q1, Q2 or Q3) will return an experiment of interest unless the numbers of cells showing nuclear-cytoplasmic translocations are low. Figure 12 shows the results of a sample search over stored data for experiments where an oscillation was observed with a period of 100 (+/- 5) minutes.

Whilst the peak detection threshold and smoothing width values have performed well with the peaks generated by protein translocations within our experimental system, it is likely that they would require adjustment for data gathered in different circumstances. Further analysis of the data we are acquiring may lead us to be able to define features within the data (such as that described in Figure 9) that will allow us to dynamically assign these parameter values whilst the algorithm is running. Future development of the algorithm and associated infrastructure will allow stored data to be re-analysed using different parameters.

## Discussion

The success of the work can be measured by how well it meets the requirements:

R1. *Logical and appropriate representation of experimental metadata and associated results*. The data model is general enough to describe a variety of microscopy experiments (fluorescence, luminescence, FISH and FRET) in both high and low content screening configurations, but it is specific enough to ensure effective validation of captured data. The model also represents the results generated by CellTracker. This may be contrasted with other data models for microscopy data (e.g. [15]) which focus on the context of an image rather than the details of the experiment and its result.

R2. *Efficient mechanisms for data capture*. Pedro has proved to be an adaptable and efficient data capture tool. By extracting details of microscope settings, locations observed under the microscope and data channels recorded from files generated by the equipment, and analysis results from the output of CellTracker, the time taken to submit a fully annotated experiment to the repository is roughly 10–15 minutes. This is very rapid when compared to the length of time it can take to annotate other functional genomics type experiments (e.g., microarrays [31]) using standard tools.

R3. *Efficient search mechanisms to identify experiments from metadata*. The model represents both the meta-data and acquired/analysed data for each experiment over which XQuery is used to implement canned queries that return results as XML documents. These documents are then formatted into appropriate reports using XSLT. As the reports are generally of similar format, implementing new queries is simply a matter of writing a new XQuery to retrieve data meeting the desired parameters.

R4. *Automated annotation of results to allow searching by result*. This has been successfully implemented and performs well with currently available data. As this feature facilitates the identification of features within data it also adds impetus for experimentalists to submit their data to the database.

The effective modelling and storage of high throughput imaging data will remain an issue as technology improves and throughput rates increase. Our repository is a fully functional example of how these data may be effectively indexed and managed to address the requirements of end users. Particularly we move away from simply providing an index of contextual information about experiments and allow this to be complemented by indexing and describing the content of data gathered in these different contexts.

The system is currently in use by a group of 10 users who are all based in the same location. However, the repository is currently housed on a central server that is accessible from any authorised (this is dependant on server configuration) computer on the internet. The client Pedro software for submitting data to the database may also be run from any machine connected to the internet. This wide accessibility enables all individuals within a group to have their own client on their own machine for data submission without having to rely on access to a specific machine and would also lend itself to situations where collaborations require individuals from multiple institutions to have access to the repository.

The novelty of our system, and a key factor in its uptake by experimentalists, has been the inclusion of the peak spotting algorithm enabling the correlation of results across disparate experiments. With many biological repositories, data entry can be time consuming and, understandably, many individuals are disinclined to partake in this activity. By adding extra value to what would otherwise be simply a metadata and results repository, there is a clear benefit for individuals to ensure that their data forms part of the searchable corpus.

Whilst other solutions currently available address the management and analysis of image data, the database presented here demonstrates a solution and model for the effective management of experimental data gathered from high content live cell imaging. Despite the system being tailored to a specific arrangement and set of techniques, the approach and model are generic enough to be applicable to other functional proteomics/genomics experimental set ups.

## Availability and Requirements

• **Project name**: livecellim

• **Project home page**: 

• **Operating system(s)**: Windows XP, Mac OS X 1.5.4 or later.

• **Programming language**: Java

• **Other requirements**: Minimum 2 GB RAM. JRE 6.0, Apache Tomcat 6.0, eXist-DB 1.2.4 or later.

• **License**: BSD

## Authors' contributions

DJ wrote the text, refined the data model, refined the data capture process and tools, and designed, implemented and tested the peak spotting algorithm. TG designed and implemented the first version of the database and data capture tools. NS wrote the servlet architecture that is used by the front end along with many utility libraries. DAT, JA, SK, and SR provided and analysed test data for the algorithm as well as contributing through many discussions to the final data model. DS and MRHW supervised the microscopy experiments. SGO, DBK and MRHW supervised the application-oriented aspects of the project, and NWP supervised the computational aspects. All authors read and approved the final manuscript.

## Supplementary Material

Additional file 1 **Installation instructions**. How to install a working instance of the image database. Click here for file

Additional file 2 **Data model XML schema**. This XSD document describes the data model used to capture the data relating to imaging experiments for the database. Click here for file

Additional file 3 **Sample experiment XML files**. This file contains both a minimal experimental annotation and a complete annotation which includes results in XML format that may be uploaded to the database. The complete.xml file in the zip archive also contains CellTracker output XML within the <Result> tags. Click here for file

Additional file 4 **Database web front end application**. The web application front end for deployment on a Tomcat server. Click here for file

Additional file 5 **Pre-configured Pedro data capture tool**. Pedro data capture tool configured to function with eXist XML database. Click here for file

Additional file 6 **Source code**. Java source code for the Pedro plugins and front end elements. Click here for file

Additional file 7 **Test and Training Data Details**. Details of protocol, cell lines and plasmids for the Test and Training Data. Click here for file

Additional file 8 **Training Data**. Spreadsheet files containing the raw Nuclear:Cytoplasmic ratios for the training data. Click here for file

Additional file 9 **Test Data**. Spreadsheet files containing the raw Nuclear:Cytoplasmic ratios for the test data. Click here for file

Additional file 10 **Raw counts**. Counts of True Positives, False Positives and False Negatives for the training and test data. Click here for file
