# Supplementary material for: Information management for high content live cell imaging
Source: BMC Bioinformatics. 2009 Jul 21;10:226. doi: 10.1186/1471-2105-10-226 (PMC2723092; doi:10.1186/1471-2105-10-226)
Supplement: Additional file 1 — Installation instructions. How to install a working instance of the image database. [file 1471-2105-10-226-S1.rtf]

Deploying a test installation of the Beacon project Image DatabaseRequirements:Sun JDK 1.6.x 		(http://java.sun.com)eXist 1.2.4			(http://exist-db.org)Apache Tomcat 6.x		(http://tomcat.apache.org/download-60.cgi)At least 2gb RAM on client machine.Steps:1. Install JDK and ensure you set the JAVA_HOME environment variable to point at the JDK installation.2. Install eXist, standalone,  using port 8080 and set up a user account with admin permissions as per the eXist documentation.3. Install Tomcat, making sure to specify a port other than 8080.4. Unzip webapp.zip to the Tomcat webapps directory and start the Tomcat server as per the Tomcat documentation.5. Edit existBeacon\WEB-INF\web.xml to specify the username and password set up in step 2.5. Unzip configuredpedro.zip to a convenient location.6. Experiment records may be edited using Pedro.  See the “Using Pedro” notes for a brief guide.7. To view uploaded records use a web browser to point at:http://localhost:PORT/existBeacon/index.jsp?application=Beacon&directory=HomeWhere PORT is the port that Tomcat is running on.This will not work if you haven't created at least one experiment document.Using PedroFor more detailed general instructions please see:http://pedrodownload.man.ac.uk/Pedro is run by the run_pedro.bat file.  It may be possible to use Pedro with Linux by editing the run_pedro.sh file as appropriate, but we make no guarantees as to compatibility. Pedro relies on information stored within the various model directories stored in “models”.  The supplied configuration assumes that eXist is running on the local machine. If you wish to use a different eXist server, you must edit the “Database.xml” file to refer to this server in the “config” directory for each model.To load an initial experiment into the database, choose the “Experiments” model from the initial Pedro menu, then from the File menu select “Load” and select one of the sample PDZ (Pedro’s native file format) files to load from the directory Pedro resides in:complete.pdz (may take some time to load but includes numerical results) or smaller.pdzThese files correspond to “complete.xml” and “small.xml” which are in the examplexml.zip file.From the plugins menu in the top right, choose “Save experiment to database.” and enter your username/password as appropriate. Assuming eXist is configured correctly the experiment file will be uploaded and committed to the database.If you wish to change elements of the experimental description, you may use the document tree on the right to navigate to the elements which you wish to change.  After having edited an item, be sure to click “Keep” at the bottom of the data entry window (you may have to scroll down to see this button).Experiments are indexed by title and start date.  Therefore, if you make changes to either of these a new experiment file will be created and saved.  Leaving these the same and updating other elements of the experiment description will overwrite the version currently in the database.Zeiss mdb files may be imported from the plugin in the “Experimental_Details” section of the input document.  Cell Tracker output may be imported from the plugin in the “Result” section of “LocationReading” document elements.
