# Supplementary material for: Information management for high content live cell imaging
Source: BMC Bioinformatics. 2009 Jul 21;10:226. doi: 10.1186/1471-2105-10-226 (PMC2723092; doi:10.1186/1471-2105-10-226)
Supplement: Additional file 5 — Pre-configured Pedro data capture tool. Pedro data capture tool configured to function with eXist XML database. [file 1471-2105-10-226-S5.zip › configuredpedro/doc/tutorials/datamodeller/ContextHelp.html]

Pedro Data Modeller Tutorial - Lessons about Data Modelling


## Pedro Tutorials

### Data Modeller Tutorials

  
Pedro Data Modeller Overview  
What Files and Where  
Context Sensitive Help  
Linking Ontologies  
Non-editable Fields  
Form Comments  
Supported XML  

### Links

  
Main Tutorial Page  
Pedro Main Page  
Contact

## Creating Context Sensitive Help Pages.

  

One of Pedro�s features is that it provides context sensitive help for the End Users. The help pages are created by the Data Modeller, usually with information provided by the domain expert for the particular data model being created.

The pages are in HTML format and may be created by any means you wish. These pages are then placed in the doc folder within the data model as mentioned above.

The help pages are then linked to the model in the ConfigurationFile in the config folder. Using the cancerPatientRecord model, an example of how to do this is:

Demographic is the name of the form on which is found the field named Ethnic\_Group for which there is a helpLink tag linking to an html page called Ethnic\_Group.html (this page will be found in the doc folder).
