# Supplementary material for: Information management for high content live cell imaging
Source: BMC Bioinformatics. 2009 Jul 21;10:226. doi: 10.1186/1471-2105-10-226 (PMC2723092; doi:10.1186/1471-2105-10-226)
Supplement: Additional file 5 — Pre-configured Pedro data capture tool. Pedro data capture tool configured to function with eXist XML database. [file 1471-2105-10-226-S5.zip › configuredpedro/doc/tutorials/datamodeller/Fields.html]

Pedro Data Modeller Tutorial - Lessons about Data Modelling


## Pedro Tutorials

### Data Modeller Tutorials

  
Pedro Data Modeller Overview  
What Files and Where  
Context Sensitive Help  
Linking Ontologies  
Non-editable Fields  
Form Comments  
Supported XML  

### Links

  
Main Tutorial Page  
Pedro Main Page  
Contact

## Making Fields Non-Editable on the Forms.

  

There may be instances where the Domain Expert wants fields to be non-editable by the End User, for example when ontology terms are the only terms acceptable for a field entry. An excerpt from the cancerPatientRecord model is below:

On the form called Prescribed\_Drug there is a field called Drug\_Name in which the End User may not write any text. The default for all fields is that they may be edited.
