# Supplementary material for: Information management for high content live cell imaging
Source: BMC Bioinformatics. 2009 Jul 21;10:226. doi: 10.1186/1471-2105-10-226 (PMC2723092; doi:10.1186/1471-2105-10-226)
Supplement: Additional file 5 — Pre-configured Pedro data capture tool. Pedro data capture tool configured to function with eXist XML database. [file 1471-2105-10-226-S5.zip › configuredpedro/doc/tutorials/datamodeller/Files.html]

Pedro Data Modeller Tutorial - Lessons about Data Modelling


## Pedro Tutorials

### Data Modeller Tutorials

  
Pedro Data Modeller Overview  
What Files and Where  
Context Sensitive Help  
Linking Ontologies  
Non-editable Fields  
Form Comments  
Supported XML  

### Links

  
Main Tutorial Page  
Pedro Main Page  
Contact

## What Files Concern the Data Modeller and Where Are They?

  

To find the folders and files needed, go to the directory sub-tree within the Pedro application where the run\_pedro batch file
is located. At this same level is a folder called models. Within this folder is where all the Data Modeller�s work is done. Going
into this folder, you will see several other folders. The names of most of these folders correspond to the data model choices that
are offered when first starting the Pedro application.

Within each of these data models is roughly the same list of folders used, among other things, to model, configure,
and store data. The folders that are common to all data models are config, doc, model, and resources. These are the folders
that come with the blankModel folder in the Pedro application download. The Pedro model comes with these and others as well many
of which are for convenience rather than necessity. The number of folders in any model depends upon the nature of the model and
what the Data Modeller feels will be of use to the End User. It is worth examining what each of these folders is meant to contain.

| Folder Name | Intended Contents |
| --- | --- |
| alerts | information relating to which alerts have been created |
| lib | executable jar files |
| src | files that are Java source code to make plugins work |
| templates | templates of parts of a model. See user tutorial for more information. |
| data | pdz files containing instances of a data model. This is where models that have been filled out are saved. |
| resources | various files used for modelling that are not the model or the config files |
| doc | html pages used for context sensitive help |
| config | the XML file for various config aspects of the data model |
| model | the data model schema in XML |

The two most important folders with which a Data Modeller will deal are the config and model folders and the files within them. These warrant closer examination.

model:

Within the model folder is the xsd file that contains the particular data model in question. In the example model of cancerPatientRecord, this file is called medinfo.xsd. This is an XML file that the Pedro application reads in order to generate the forms seen on the application. The nesting of elements corresponds to the nesting of fields on the forms. The layout of the forms is guided by the domain expert. The field types (eg. string, date, decimal, etc.) are also determined by the domain expert.

config:

The config folder can have several files in it. The only file required in all cases is the XML file Configuration.xml. It is here that several things can be done to enhance the forms used with the Pedro application. This file must make reference to the top level element in the schema.
