# Supplementary material for: Information management for high content live cell imaging
Source: BMC Bioinformatics. 2009 Jul 21;10:226. doi: 10.1186/1471-2105-10-226 (PMC2723092; doi:10.1186/1471-2105-10-226)
Supplement: Additional file 5 — Pre-configured Pedro data capture tool. Pedro data capture tool configured to function with eXist XML database. [file 1471-2105-10-226-S5.zip › configuredpedro/doc/tutorials/datamodeller/Linking.html]

Pedro Data Modeller Tutorial - Lessons about Data Modelling


## Pedro Tutorials

### Data Modeller Tutorials

  
Pedro Data Modeller Overview  
What Files and Where  
Context Sensitive Help  
Linking Ontologies  
Non-editable Fields  
Form Comments  
Supported XML  

### Links

  
Main Tutorial Page  
Pedro Main Page  
Contact

## Linking Ontologies to the Data Model.

  

There is still much debate over the exact definition of an ontology. For the purposes of this tutorial, an ontology can be thought of as a pre-determined arrangement of controlled terminology and vocabulary for use in better describing a term. This is perhaps a pragmatic definition but Pedro is a tool and not an unreliable one.

The Data Modeller would be expected to work with the Developer to get ontology services set up. From the cancerPatientRecord model, here�s what it looks like:

The actual rendering of the ontology terms will depend on how many of them there are. Just a few terms will result in a straight list; more than a few and Pedro will attempt to group them into sections; more than 40 terms and a separate browse window will appear.

Pedro comes with a few ontology source classes that parse common file formats. These are:

| Ontology Source | Description |
| --- | --- |
| SingleColumnTextSource | parses a simple text file whose terms are in a column. |
| TabIndentedSource | parses tab delimited text files where tabs represent levels in a tree hierarchy of terms. |
| XMLOntologySource | parses a simple bespoke XML format for specifying ontology terms. You can associate each ontology term with a text definition and a web page. |
| PictureOntologySource | extends the functionality of XMLOntologySource by also allowing thumbnail pictures to be associated with ontology terms. For examples of how to write this file format, please download the "ontology" model from pedrodownloac.man.ac.uk |
