# Supplementary material for: Information management for high content live cell imaging
Source: BMC Bioinformatics. 2009 Jul 21;10:226. doi: 10.1186/1471-2105-10-226 (PMC2723092; doi:10.1186/1471-2105-10-226)
Supplement: Additional file 5 — Pre-configured Pedro data capture tool. Pedro data capture tool configured to function with eXist XML database. [file 1471-2105-10-226-S5.zip › configuredpedro/doc/tutorials/datamodeller/StartingTutorial.html]

Pedro Data Modeller Tutorial - Lessons about Data Modelling


## Pedro Tutorials

### Data Modeller Tutorials

  
Pedro Data Modeller Overview  
What Files and Where  
Context Sensitive Help  
Linking Ontologies  
Non-editable Fields  
Form Comments  
Supported XML  

### Links

  
Main Tutorial Page  
Pedro Main Page  
Contact

## Data Modeller Tutorial - Learning About Pedro and Data Models

  

The Data Modeller is defined in the Pedro context as someone who creates the forms that are seen on the application. While the Data Modeller and the End User may be the same person, the roles are in fact different. The Data Modeller is expected to be comfortable with the technical aspects of the Pedro tool, such as XML, more so than the End User. However, the Data Modeller is not expected to programme in the underlying application code. The Data Modeller is responsible for rapid prototyping of forms and the creation of schemas. Practice has shown that the Data Modeller need not be a domain expert to create data models for specific domains.
