# Supplementary material for: Information management for high content live cell imaging
Source: BMC Bioinformatics. 2009 Jul 21;10:226. doi: 10.1186/1471-2105-10-226 (PMC2723092; doi:10.1186/1471-2105-10-226)
Supplement: Additional file 5 — Pre-configured Pedro data capture tool. Pedro data capture tool configured to function with eXist XML database. [file 1471-2105-10-226-S5.zip › configuredpedro/doc/tutorials/datamodeller/Supported.html]

Pedro Data Modeller Tutorial - Lessons about Data Modelling


## Pedro Tutorials

### Data Modeller Tutorials

  
Pedro Data Modeller Overview  
What Files and Where  
Context Sensitive Help  
Linking Ontologies  
Non-editable Fields  
Form Comments  
Supported XML  

### Links

  
Main Tutorial Page  
Pedro Main Page  
Contact

## XML Features that are Known to be Supported in Pedro.

  

The Pedro application was initially developed to be a generic tool for rapid data modelling for the proteomics community. If you don�t know what proteomics is, don�t worry. Despite the generic approach, the proteomics model does not use any intricate or elaborate features of XML. As such

The element types that are know to be supported in Pedro are decimal, string, anyURI, date, boolean, and double. Some tags that are supported include  and .
