# Supplementary material for: Information management for high content live cell imaging
Source: BMC Bioinformatics. 2009 Jul 21;10:226. doi: 10.1186/1471-2105-10-226 (PMC2723092; doi:10.1186/1471-2105-10-226)
Supplement: Additional file 5 — Pre-configured Pedro data capture tool. Pedro data capture tool configured to function with eXist XML database. [file 1471-2105-10-226-S5.zip › configuredpedro/doc/tutorials/developer/DeveloperTutorial.html]

Developer Tutorial


|  |
| --- |
| Main Tutorial Page |

# Developer Tutorial

We assume that you know how to program in Java. In particular, you
should know how to:

- add jar files and directories to your class path
- use the java jar utility
- know about Java interfaces

### A Word about the Demo Classes

SamplePlantValidator and PlantWordSource rely on a mysql database
called "plantExperiments". The data folder has been included in the
tutorial/resources directory for your use. If that somehow doesn't
work out,  quickly construct it yourself.

In a later release, we will have fully described java docs. In the
meantime, we will describe the Pedro interfaces you can use here.
Make sure that the "pedro.jar" file found in pedro/lib is included in
your classpath.

Normally the "lib" file of your model folder should only contain jar
files. However, in pedro/dist/models/tutorial/lib, you will also find
a bundle of source code for sample classes that implement Pedro interfaces.

#### Step 1: Include pedro.jar in Your ClassPath

Include the "pedro.jar" file located in pedro/dist/lib in your
classpath. Your development environment will have to know about the
pedro interface classes.

#### Step 2: Write a Class that Implements an Interface

You should decide what you'd like to write.

#### Implementing a Validator

Validators are applied to text and date fields. Validator classes
implement the pedro.validation.Validator interface:

```
package pedro.validation;

public interface Validator {
   public String validate(String value);
   public void setFieldName(String fieldName);
   public void setRequiredField(boolean isFieldRequired);
}
```

Please take a look at
pedro/dist/models/tutorial/lib/PlantSampleValidator.java. It is a
validator that takes the value from a "sample\_code" field of an
"Experiment" record and determines whether the code already appears in
a database.

Pedro's source code isn't fully documented but is included in the
download. Navigate to "pedro/src/validation" and observe how most of
the classes inherit from an "AbstractValidator" convenience class.
Feel free to use it!

PlantSampleValidator depends on the "mm.jar" library for database
connection activities. This jar file must therefore be included in
the distribution and appears in the "lib" directory.

#### Implementing an OntologySource

Ontology Sources are responsible for providing vocabulary terms to an
Ontology Viewer, which then makes them available to the user.
Ontology sources implement "pedro.ontology.OntologySource".

Some ontology sources are capable of expressing their data as a tree
of terms. If your ontology can do this, please use
"pedro.ontology.TreeOntologySource"

The basic approach involves writing a class that implements one of
these four interfaces:

- pedro.validation.Validator
- pedro.ontology.OntologySource
- pedro.ontology.TreeOntologySource
- pedro.ontology.OntologyViewer

You make a jar file for these files and you

#### Jar your files

Bundle your files into a jar file. Then place this file in the "lib"
directory of your model folder. For example, in the tutorial file,
bundled class files are put into "myown.jar", which is in
tutorial/lib.
