# Supplementary material for: Information management for high content live cell imaging
Source: BMC Bioinformatics. 2009 Jul 21;10:226. doi: 10.1186/1471-2105-10-226 (PMC2723092; doi:10.1186/1471-2105-10-226)
Supplement: Additional file 5 — Pre-configured Pedro data capture tool. Pedro data capture tool configured to function with eXist XML database. [file 1471-2105-10-226-S5.zip › configuredpedro/doc/tutorials/developer/OntologySource.html]

Pedro Developer Tutorial - Developing With Pedro


## Pedro Tutorials

### Developer Tutorials

  
Pedro Developer Overview  
  

### Implementing Interfaces

  
OntologySource  
TreeOntologySource  
  

### Links

  
Main Tutorial Page  
Pedro Main Page  
Contact

## Implementing the OntologySource Interface

  

Ontology Sources are responsible for providing vocabulary terms to an
Ontology Viewer, which then makes them available to the user.
Ontology sources implement the following interface.

OntologySource describes a class that can generate a collection of
vocabulary terms. An ontology source provides terms to an
OntologyViewer, which then renders them for the user.

  

**Interface**  
  

```
package pedro.ontology;

import java.io.Serializable;

public interface OntologySource extends Serializable {
   public String getName();
   public String getDescription();
   public OntologyTerm[] getTerms();
   public OntologyTerm[] getRelatedTerms(OntologyTerm ontologyTerm);
   public boolean containsTerm(OntologyTerm ontologyTerm);
   public void setFileName(String fileName);
   public boolean isWorking();
   public String test();
   public OntologySource getView(String parameters);
}
```

"getView" is a method some ontology sources use to derive smaller
views of the ontology. This method is used more by
TreeOntologySources that inherit from this interface.

Note that a vocabulary word is not considered a string, but a class
that has notions of a value, an identifier and a URL. OntologyTerm is
described in the pedro/src/ontology directory. The set/get methods
for id and URL are optional. We'll be making more use of these
methods in a future release that tries to link the terms to web pages
containing a definitions.

  

**Pedro Classes Implementing OntologySource**  
  
Pedro has one class that implements this interface. The class
pedro.ontology.SingleColumnTextSource reads a text file containing a single
column of terms.

The basic approach involves writing a class that implements one of
these four interfaces:

- pedro.validation.Validator
- pedro.ontology.OntologySource
- pedro.ontology.TreeOntologySource
- pedro.ontology.OntologyViewer

You make a jar file for these files and bundle your files into this jar file. Then place this file in
the "lib" directory of your model folder.
