# Supplementary material for: Information management for high content live cell imaging
Source: BMC Bioinformatics. 2009 Jul 21;10:226. doi: 10.1186/1471-2105-10-226 (PMC2723092; doi:10.1186/1471-2105-10-226)
Supplement: Additional file 5 — Pre-configured Pedro data capture tool. Pedro data capture tool configured to function with eXist XML database. [file 1471-2105-10-226-S5.zip › configuredpedro/doc/tutorials/developer/OntologySourceOld.html]

Interface: OntologySource


|  |
| --- |
| Developer Tutorial Page |

# Interface: OntologySource

OntologySource describes a class that can generate a collection of
vocabulary terms. An ontology source provides terms to an
OntologyViewer, which then renders them for the user.

### Interface

```
package pedro.ontology;

import java.io.Serializable;

public interface OntologySource extends Serializable {
   public String getName();
   public String getDescription();
   public OntologyTerm[] getTerms();
   public OntologyTerm[] getRelatedTerms(OntologyTerm ontologyTerm);
   public boolean containsTerm(OntologyTerm ontologyTerm);
   public void setFileName(String fileName);
   public boolean isWorking();
   public String test();
   public OntologySource getView(String parameters);
}
```

"getView" is a method some ontology sources use to derive smaller
views of the ontology. This method is used more by
TreeOntologySources that inherit from this interface.

Note that a vocabulary word is not considered a string, but a class
that has notions of a value, an identifier and a URL. OntologyTerm is
described in the pedro/src/ontology directory. The set/get methods
for id and URL are optional. We'll be making more use of these
methods in a future release that tries to link the terms to web pages
containing a definitions.

### Pedro Classes Implementing OntologySource

Pedro has one class that implements this interface. The class
pedro.ontology.SingleColumnTextSource reads a text file containing a single
column of terms.

### Example Ontology Source

See pedro/dist/models/tutorial/dist/lib/PlantWordSource.java for an
example of a class that implements OntologySource. It gets its terms
from a table in a database. The class depends on "mm.jar", so the jar
file is included in the "lib" directory.
