# Supplementary material for: Information management for high content live cell imaging
Source: BMC Bioinformatics. 2009 Jul 21;10:226. doi: 10.1186/1471-2105-10-226 (PMC2723092; doi:10.1186/1471-2105-10-226)
Supplement: Additional file 5 — Pre-configured Pedro data capture tool. Pedro data capture tool configured to function with eXist XML database. [file 1471-2105-10-226-S5.zip › configuredpedro/doc/tutorials/developer/PlantExperiment.html]

Constructing the Plant Experiment Database


|  |
| --- |
| Developer Tutorial Page |

# Constructing the Plant Experiment Database

Make sure you've got mySQL set up. Create a database called
"plantExperiments". Add these tables to it.

### Table: "samples"

|  |  |
| --- | --- |
| **Field** | **Type** |
| id | varchar(20) |

### "Samples" Sample Data

FL-332, FL-333, FL-334, FL-335.

If you get this working, then Pedro should signal an error if you
enter any of these values.

### Table: "vocabulary"

|  |  |
| --- | --- |
| **Field** | **Type** |
| term | varchar(50) |

### "Vocabulary" Sample Data

potato, leek, carrot, celery, parsnip.

OK, this data is a bit nonsensical. The point is can you pull these
terms into Pedro?
