# Supplementary material for: Information management for high content live cell imaging
Source: BMC Bioinformatics. 2009 Jul 21;10:226. doi: 10.1186/1471-2105-10-226 (PMC2723092; doi:10.1186/1471-2105-10-226)
Supplement: Additional file 5 — Pre-configured Pedro data capture tool. Pedro data capture tool configured to function with eXist XML database. [file 1471-2105-10-226-S5.zip › configuredpedro/doc/tutorials/developer/StartingTutorial.html]

Pedro Developer Tutorial - Developing With Pedro


## Pedro Tutorials

### Developer Tutorials

  
Pedro Developer Overview  
  

### Links

  
Main Tutorial Page  
Pedro Main Page  
Contact

## Developer Tutorial - Some Aspects of Developing with Pedro

  

Doing development with Pedro requires a knowledge of Java. In particular, you
should know how to:

- add jar files and directories to your class path
- use the java jar utility
- know about Java interfaces

In a later release, we will have fully described java docs. In the
meantime, we will describe the Pedro interfaces you can use here.
Make sure that the "pedro.jar" file found in pedro/lib is included in
your classpath.

**Step 1: Include pedro.jar in Your ClassPath**  
  
Include the "pedro.jar" file located in pedro/dist/lib in your
classpath. Your development environment will have to know about the
pedro interface classes.

  

**Step 2: Write a Class that Implements an Interface**  
  
You should decide what you'd like to write. You would create a java
class that implemented one or more of the following interfaces. The
methods for these interfaces are well described in the source code
bundle that comes with the distribution.

The interfaces:

|  |  |
| --- | --- |
| pedro.validation.Validator | Lets you create your own customised field validation routines. For example, you could write an implementation that looks up a field value in a database of unique keys. It could generate an error if the field value already appeared in the database. When you finish your implementation of the Validator interface, you can reference it using the "validator" XML tag in the ConfigurationFile.xml. The contents of this tag will be the fully qualified path name of the validation class you created. |
| pedro.ontology.sources.OntologySource | Lets you create your own supplier of ontology terms. For example, you may store a controlled vocabulary in some particular file format. Your ontology source class would probably reference some parser program you use to read the file of ontology terms. You would then implement various methods in OntologySource that would allow the class to supply terms to the rest of the Pedro system. An example of an ontology source class is pedro.ontology.sources.SingleColumnTextSource. To link ontology sources with form fields please read part of the data modeller tutorial |
| pedro.ontology.sources.TreeOntologySource | This extends the functionality of OntologySource but allows your source to supply terms in the form of a tree. Example implementation of this interface are pedro.ontology.sources.TabIndentedSource and pedro.ontology.sources.XMLOntologySource. To link ontology sources with form fields please read part of the data modeller tutorial |
| pedro.ontology.sources.URLDescriptionSupport | This is a marker interface that has no methods. Pedro has its own default way of rendering the terms supplied by an ontology source. It analyses what interfaces your class implements in order to make decisions about rendering the terms to the user. All ontology sources can supply their terms in the form of a list. If the class implements TreeOntologySource, the application knows it can allow users to use a tree display to find the terms they want. If the class implements URLDescriptionSupport, it means that for most requests, Pedro can expect a call to "getWebPage(...)" to come back with a result. The application will then show a viewer that has a window pane for showing HTML pages. |
| pedro.ontology.sources.DictionaryDescriptionSupport | This is a marker interface that Pedro can use to decide how best to render the collection of terms provided by the ontology source. A class that implements OntologySource as well as DictionaryDescriptionSupport will in most cases return a value for the method "getDefinition(...)". This means Pedro's default viewer will show a table view with the first column representing terms and the second column representing term definitions. |
| pedro.ontology.sources.ImageDescriptionSupport | This is an interface that Pedro can use to decide how best to render the collection of terms provided by the ontology source. Pedro has a default view for ontology sources that are capable of supplying an image for each term. You would make your ontology source class also implement this interface and you would write an implementation of the: "public Image getImage(...)" method. |
| pedro.ontology.views.OntologyViewer | allows you to use your own software component for rendering ontology terms. For example, you may have a display that can render a collection of ontology terms as a graph. You could make your viewer implement methods of OntologyViewer, then use it to visualise terms provided by various ontology sources. You may also use this class if you are trying to integrate an existing viewer application with the application. You would have to find some way of gathering selected ontology terms and inserting them into the form field that called the viewer. If you need help making your own ontology viewer, consult pedro.views.DefaultOntologyViewer. To link ontology sources with form fields please read part of the data modeller tutorial |
|

**Step 3: Create a JAR file that bundles the files you've
created.**  
  
You need to package the \*.class files that are created by compiling
your new \*.java files.

**Step 4: Put the JAR file in a location where Pedro can read it** 
Put the JAR file in pedro/dist/*myModel*/lib where "myModel" is the
name of the model folder you're working on. When you start the Pedro application, it
will load all the JAR files you've placed in the "lib" directory of
your model folder.
