# Supplementary material for: Information management for high content live cell imaging
Source: BMC Bioinformatics. 2009 Jul 21;10:226. doi: 10.1186/1471-2105-10-226 (PMC2723092; doi:10.1186/1471-2105-10-226)
Supplement: Additional file 5 — Pre-configured Pedro data capture tool. Pedro data capture tool configured to function with eXist XML database. [file 1471-2105-10-226-S5.zip › configuredpedro/doc/tutorials/developer/TreeOntologySource.html]

Pedro Developer Tutorial - Developing With Pedro


## Pedro Tutorials

### Developer Tutorials

  
Pedro Developer Overview  
  

### Implementing Interfaces

  
OntologySource  
TreeOntologySource  
  

### Links

  
Main Tutorial Page  
Pedro Main Page  
Contact

## Implementing the TreeOntologySource Interface

  

Some ontology sources are capable of expressing their data as a tree
of terms. If your ontology can do this, please use the interface below.
This interface extends OntologySource by letting the implementer express the ontology as a tree
of nodes.

  

**Interface**  
  

```
package pedro.ontology;

import java.io.Serializable;

public interface TreeOntologySource extends OntologySource {
   public OntologyTermNode getTreeRoot();
}
```

"OntologyTermNode" is described in the pedro/src/ontology source code
directory. The class simply extends DefaultMutableTreeNode, but
allows an OntologyTerm instead of a String to passed to the constructor.

  

**Pedro Classes Implementing TreeOntologySource**  
  
Pedro has two classes that implement this interface.
pedro.ontology.TabIndentedTextSource and pedro.ontology.XMLOntologySource both extend
pedro.ontology.AbstractTreeOntologySource, which implements the TreeOntologySource
interface.

The basic approach involves writing a class that implements one of
these four interfaces:

- pedro.validation.Validator
- pedro.ontology.OntologySource
- pedro.ontology.TreeOntologySource
- pedro.ontology.OntologyViewer

You make a jar file for these files and bundle your files into this jar file. Then place this file in
the "lib" directory of your model folder.
