# Supplementary material for: Information management for high content live cell imaging
Source: BMC Bioinformatics. 2009 Jul 21;10:226. doi: 10.1186/1471-2105-10-226 (PMC2723092; doi:10.1186/1471-2105-10-226)
Supplement: Additional file 5 — Pre-configured Pedro data capture tool. Pedro data capture tool configured to function with eXist XML database. [file 1471-2105-10-226-S5.zip › configuredpedro/doc/tutorials/developer/TreeOntologySourceOld.html]

Interface: TreeOntologySource


|  |
| --- |
| Developer Tutorial Page |

# Interface: TreeOntologySource

This interface extends Ontology
Source by letting the implementer express the ontology as a tree
of nodes.

### Interface

```
package pedro.ontology;

import java.io.Serializable;

public interface TreeOntologySource extends OntologySource {
   public OntologyTermNode getTreeRoot();
}
```

"OntologyTermNode" is described in the pedro/src/ontology source code
directory. The class simply extends DefaultMutableTreeNode, but
allows an OntologyTerm instead of a String to passed to the constructor.

### Pedro Classes Implementing OntologySource

Pedro has two classes that implement this interface.
pedro.ontology.TabIndentedTextSource and pedro.ontology.XMLOntologySource both extend
pedro.ontology.AbstractTreeOntologySource, which implements the TreeOntologySource
interface.

### Example Ontology Source

The best examples are the ones Pedro already uses. Please scan
through the pedro/src/ontology directory.
