# Supplementary material for: Information management for high content live cell imaging
Source: BMC Bioinformatics. 2009 Jul 21;10:226. doi: 10.1186/1471-2105-10-226 (PMC2723092; doi:10.1186/1471-2105-10-226)
Supplement: Additional file 5 — Pre-configured Pedro data capture tool. Pedro data capture tool configured to function with eXist XML database. [file 1471-2105-10-226-S5.zip › configuredpedro/doc/tutorials/MainTutorial.html]

Pedro Tutorials - Main


## Pedro Tutorials

### Pedro Tutorials - Main

  
Pedro Tutorial Overview  
User Tutorial  
Data Modeller Tutorial  
Plugins Tutorial  
Developer Tutorial  
  

### Links

  
Pedro Main Page  
Contact

## Pedro Tutorial Overview

  

### What can Pedro do?

This will, hopefully, be the first page you've come across to find out about Pedro. It is worth starting with a quick run down of what this tool is about and what it can do. Pedro is a downloadable Java application that allows for rapid prototyping of data models based upon an XML schema. The aspects of the data models take the appearance of data entry forms. The logical extension of the data modelling is thus data entry. Pedro is particularly useful if the schema you are currently using is in flux or is anticipated to change eventually to as yet undefined

Pedro was originally developed for the proteomics community and we
ship their data model for those scientists to use. To Pedro however, the data model
could be about any field or discipline that uses an XML schema. In this way the Pedro application adheres to a generic architecture. Throughout the tutorials we have tried to use a particular model called cancerPatientRecord which, as the name implies, is based on a mock model from the health care domain.

### The Kinds of Tutorials

User Tutorial:

This tutorial is for people using Pedro primarily as a data entry tool. The expectation is that these people will probably not have a lot of technical experience or expertise. We've tried to make the aspects of this tutorial as free of technical jargon as possible and you will find many example screen shots of what the outcome of your entries might look like and how to make changes.

Data Modeller Tutorial:

This tutorial is for people doing rapid data modelling. There is an expectation that these people are perhaps more technical than an end user. Data modellers should be familiar with XML and files/directory structures. Practice has shown that a data modeller need not be a domain expert for the particular model being created. There is no need for the data modeller to be familiar with Java.

Plugins Tutorial:

This tutorial is for the group of people who may want to develop plugins for Pedro. While these people may be quite technical, there is no expectation that they are familiar with the inner workings of the Pedro application.
