# Supplementary material for: Information management for high content live cell imaging
Source: BMC Bioinformatics. 2009 Jul 21;10:226. doi: 10.1186/1471-2105-10-226 (PMC2723092; doi:10.1186/1471-2105-10-226)
Supplement: Additional file 5 — Pre-configured Pedro data capture tool. Pedro data capture tool configured to function with eXist XML database. [file 1471-2105-10-226-S5.zip › configuredpedro/doc/tutorials/plugins/MoreDetails.html]

Pedro User Tutorial - Lessons about Data Entry


## Pedro Tutorials

### Plugins Tutorial

  
Writing Plugins for Pedro  
More Details of Plugins  

### Links

  
Main Tutorial Page  
Pedro Main Page  
Contact

## More Details on Pedro Plugins

  

Because we're trying to get this to release quickly some short cuts will be taken here. The files you're going to want are located in the
source directory under pedro/plugins/.... . Some Java files have been included here to show you what they might look like: MassSpecExperimentLoader.java, PeakListAnalysis.java, PeakListExporter.java, and PeakListValidation.java.
The main classes you'll have to be concerned with:

- using pedro.model.RecordModelFactory to instantiate new instances of
  records of type X;
- pedro.model.RecordModel, pedro.model.ListFieldModel and
  pedro.model.EditFieldModel, which are the basic elements that create
  Pedro's tree of native data structures;
- use the RecordModel methods to set or get particular
  fields. You then set or get the value of these fields;
- pedro.view.NavigationTree, where you may make use of the
  pasteNode(...) method to overwrite a blank record with records you
  create from applying a data import method.

So let's say you want to create a data import plugin. You're going to
create a subtree of new records of types X,Y,Z. You use the
RecordModelFactory class to create blank versions of these record
types. Then you interrogate the RecordModel, ListFieldModel and
EditFieldModels to stuff data into the structures. At the end you
invoke the NavigationTree's pasteNode method so it will appear in the
tree.

Please take a look at the Java files, especially MassSpecExperimentLoader.java.
