# Supplementary material for: Information management for high content live cell imaging
Source: BMC Bioinformatics. 2009 Jul 21;10:226. doi: 10.1186/1471-2105-10-226 (PMC2723092; doi:10.1186/1471-2105-10-226)
Supplement: Additional file 5 — Pre-configured Pedro data capture tool. Pedro data capture tool configured to function with eXist XML database. [file 1471-2105-10-226-S5.zip › configuredpedro/doc/tutorials/plugins/WritingPlugins.html]

Pedro User Tutorial - Lessons about Data Entry


## Pedro Tutorials

### Plugins Tutorial

  
Writing Plugins for Pedro  
More Details of Plugins  

### Links

  
Main Tutorial Page  
Pedro Main Page  
Contact

## Writing Pedro Plugins

  

This section is intended for the developers in early adopter groups.
The goal here is to allow people to begin writing their own program
modules that perform data import, data export, validation or analysis
on a subtree of records beginning at a root record of type X. Here
are some plugin ideas; for now most of these will mean something to
proteomics scientists. In the near future this documentation will be
greatly improved. Examples:

- You want to populate a blank record "MASS\_SPEC\_EXPERIMENT" and all its
  subrecords using a special import routine;
- You want to export values in a record subtree to a database beginning
  at some root record;
- You want to scan a record subtree starting at a record of type X (say
  "Experimeent") to see whether there are any patterns of filling in
  experiment parts that represent poor patterns of experiment design;
- You want to some analysis on all the Peak values in a Peak List.

If you aren't in proteomics, don't worry. Your steps as a developer
are going to be fairly straight forward:

1. Create a Java class that implements one of these interfaces:
   - pedro.plugins.DataImportPlugin
   - pedro.plugins.DataExportPlugin
   - pedro.plugins.ValidationPlugin
   - pedro.plugins.AnalysisPlugin
2. Create a jar file for the plugin classes you make. We're going to
   assume for now you know how to use the Java Jar utility. As a quick
   reminder, the usual way of invoking it looks something like this: "jar cvf myplugin.jar ./myplugins";
3. rename the file so it ends with ".plugins" instead of ".jar";
4. put it in the "model/lib" directory;
5. Load the Pedro data capture tool with the appropriate model. Pedro
   loads treats all files ending in .jar or .plugins as JAR files that
   contain class files. It specifically looks in .plugins files for
   those classes that will implement one of the plugin interfaces already
   mentioned.

   These plugins are instantiated and held in memory. When the end-user
   moves from one record to the next, Pedro asks its collection of
   plugins can deal with Record type X as it is defined in the current
   record model version Y.

   As a plugin developer you can make the plugin respond in any way you
   want. If the plugin agrees it can do something with it, then it
   appears in the Services menu.

   If at least one plugin can do something the menu glows red and the
   status bar shows the kinds of plugins available.
6. That's it! This can now be revisited in more detail.
