# Supplementary material for: Information management for high content live cell imaging
Source: BMC Bioinformatics. 2009 Jul 21;10:226. doi: 10.1186/1471-2105-10-226 (PMC2723092; doi:10.1186/1471-2105-10-226)
Supplement: Additional file 5 — Pre-configured Pedro data capture tool. Pedro data capture tool configured to function with eXist XML database. [file 1471-2105-10-226-S5.zip › configuredpedro/doc/tutorials/user/Alerts.html]

Pedro User Tutorial - Lessons about Data Entry


## Pedro Tutorials

### User Tutorials

  
Pedro User Tutorial Overview  
Parts of a Pedro Window   
File Management  
File Editing  
Templates  
Importing Data  
Backup Files  
Viewing  
Searching  
Ontologies  
Context Help  
Exporting Files  
Alerts  
  
  

### Links

  
Main Tutorial Page  
Pedro Main Page  
Contact

## Alerts

  

### Learn how to ...

- create alerts;
- use alerts.

The Pedro Alerts feature is a new feature we would like to test with
the user community. The alerts system enhances Pedro's ability to
check your data files for errors and to allow scientists to discover
who else might be interested in their work.

### What is a Pedro Alert?

An **alert** is a set of matching criteria for a given record type
that is associated with one of four intents:

- an error;
- a warning;
- an information bulletin;
- a request for contact.

As an example, suppose a data model has a record called "Specimen"
which has the following fields:

- species;
- category;
- anatomy\_part.

We could make an alert with these matching criteria:

- category="invertebrate";
- anatomy\_part="spine".

Using the editor, we can associate these criteria with an error
intent: by definition, invertebrates cannot have spines.

Another alert could have these matching criteria:

- species="cyanea capillata";
- category="jellyfish";
- anatomy\_part="tentacles".

We could associate these criteria with a request for contact intent.
This would mean that if a record of type "Specimen" had these matching
field values, it would be of interest to some other user.

The following screen shot shows what an alert for proteomics might look like:

The matching criteria appear in the lower part of the right-hand
portion of the editor. The intent type here is an Information Alert.
The fields in the top right part of the right panel describe the
message the author wants to convey to other scientists whose work
fits with the matching criteria.

Scientists in one lab can use the Pedro Alerts Editor to create a
collection of alerts called an **alert bundle**. The alert bundle is a
zip file that can store descriptions of multiple alerts such as the
error relating to invertebrates and the request for contact relating
to cyanea capillata specimens.

Other scientists using Pedro can optionally include the alert bundle
in the process the tool uses to validate data files. At a minimum,
Pedro performs checks on every record in the current file to ensure it
doesn't violate properties described by the XML Schema. Errors could
include situations where the user has forgotten to fill in a required
subrecord or has typed letters in some numeric field present on the
current form.

A scientist in one lab uses Pedro's Alerts editor to create a
collection of Pedro alerts called an Alert Bundle. When users
validate their data files, they can reference the bundle through a
program feature. During validation, the application scans the records
for whether any of them match any conditions specified by a Pedro
Alert. Those that do will appear in a validation report that will
list errors, warnings, information bulletins and requests for contact.

### What will alert bundles allow users to do?

The Pedro Alerts System allows laboratories to create their own "tips
and tricks" for data entry that can be used by others when they either
explicitly try to validate their data files or when they attempt to
export them for submission to a data repository.

In older versions of the tool, Pedro's validation facilities were
limited to situations where the file was incomplete or where an
illegal value appeared in a data field. With the new alerts system,
combinations of field values within the same record can be flagged as
an error, a warning or conditions relevant to an information bulletin or
to a request for further collaboration.

The alerts system allows scientists to communicate with each other in
a way that doesn't intrude on their work. You won't be automatically
e-mailed if you validate a file and it contains some features that are
of interest to another scientist. The use of an alerts bundle is
optional so you can exclude a bundle if you don't agree with advice
supplied by the authors.

### Creating Alerts

1. Double click on the "run\_alerts\_editor.bat" file located in the
   pedro/dist directory.
2. If you have more than one model folder
   installed in the pedro/dist/models folder you may be asked to choose
   which model you want to load. If you see this dialog, choose which
   model you want to use and press "OK". For the purposes of this
   tutorial, select the pedro data model.
3. You should now see the dialog shown in the figure below. To create a
   new alert, press one of "Create Error Alert", "Create Warning Alert",
   "Create Information Alert" or "Create Contact Alert" buttons.
4. Fill in the top fields of the alert. Remember that these will later
   be displayed to other users who encounter them in their files.
5. Establish the matching criteria. From the "Select" drop down box,
   select the type of record structure you want to use.
6. Decide whether you want to add an edit field criterion or a list field
   criterion and press the appropriate button under the "Select" drop
   down box. Remember that an edit field will contain some kind of value
   such as a radio selection choice or a text entry. A list field
   contains other records.
7. If you select "Add Edit Field Criterion...", a row in the criteria
   table should appear. From left to right, the fields are: field name,
   condition and value.
8. If you select "Add List Field Criterion...", a row in the criteria
   table should appear. From left to right, the fields are: field name,
9. The following picture shows what a completed Pedro Alert might look
   like. When you have finished writing an alert, press the "Save"
   button in the lower right hand corner.- You could create several alerts in the same bundle. When you're
     finished making your alert bundle, click on the "File" menu, then press
     the "Save As..." button. A dialog will prompt you to save the file.
   - Alert bundles are stored as .zip files. It's a good idea to post the
     zip file on a web page or at some regular known location so other
     users can easily reference it from their own sessions of Pedro.

### Using Alerts

1. Run the Pedro data capture tool using the Pedro data model.
2. Click the "Options" menu, then the "Alerts..." option.
3. You should now see the Alert Bundle Selection Dialog.

   You can specify
   a URL for the .zip alert bundle or browse for the file by pressing the
   "Browse" button. In the release, check inside the pedro/alerts
   subfolder.
4. When you have specified a URL or file, click the
   "Add" button. The file or URL should appear in the list of selected
   alert bundles.
5. You may want to inspect the bundles to make sure you agree with the
   advice they provide. Select a bundle and press the "View" button. An
   Alerts Viewer dialog should appear. When you're done inspecting the alerts, press the dialog's "Close" button.
6. There are two situations where the alert bundles will be applied:
   - when you click the "View" menu, then the "Show Errors" option.
   - when you click the "File" menu, then the "Export to Final Submission
     Format" button.
7. If errors are found during validation, the following dialog will appear:
