# Supplementary material for: Information management for high content live cell imaging
Source: BMC Bioinformatics. 2009 Jul 21;10:226. doi: 10.1186/1471-2105-10-226 (PMC2723092; doi:10.1186/1471-2105-10-226)
Supplement: Additional file 5 — Pre-configured Pedro data capture tool. Pedro data capture tool configured to function with eXist XML database. [file 1471-2105-10-226-S5.zip › configuredpedro/doc/tutorials/user/BackupFiles.html]

Pedro User Tutorial - Lessons about Data Entry


## Pedro Tutorials

### User Tutorials

  
Pedro User Tutorial Overview  
Parts of a Pedro Window   
File Management  
File Editing  
Templates  
Importing Data  
Backup Files  
Viewing  
Searching  
Ontologies  
Context Help  
Exporting Files  
Alerts  
  
  

### Links

  
Main Tutorial Page  
Pedro Main Page  
Contact

## Backup Files

  

### Learn how to ...

- use backup files.

### Using Backup Files

Each time you press **Save** from the **File** option of the menu bar, Pedro saves
a backup file. Pressing **Save** produces a two files, each with a slightly different file extension; the regular Pedro file name ends with *.pdz* while the backup file name ends with *.pdz~*. If you want to see the data file as it was before you last pressed **Save**, you can load the backup file by opening it the way you would any file.
