# Supplementary material for: Information management for high content live cell imaging
Source: BMC Bioinformatics. 2009 Jul 21;10:226. doi: 10.1186/1471-2105-10-226 (PMC2723092; doi:10.1186/1471-2105-10-226)
Supplement: Additional file 5 — Pre-configured Pedro data capture tool. Pedro data capture tool configured to function with eXist XML database. [file 1471-2105-10-226-S5.zip › configuredpedro/doc/tutorials/user/ContextHelp.html]

Pedro User Tutorial - Lessons about Data Entry


## Pedro Tutorials

### User Tutorials

  
Pedro User Tutorial Overview  
Parts of a Pedro Window   
File Management  
File Editing  
Templates  
Importing Data  
Backup Files  
Viewing  
Searching  
Ontologies  
Context Help  
Exporting Files  
Alerts  
  
  

### Links

  
Main Tutorial Page  
Pedro Main Page  
Contact

## Context Help

  

### Learn how to ...

- get information about terms in the model.

It may not always be clear what certain terms in a given model mean. Pedro allows users to access information about terms that have been described by the data modeller. This feature is defaulted to off when Pedro starts up. To turn this feature on click on **Help** on the menu bar and then select **Enable context help**. If you then repeat this, there should be a check mark in the box just next the **Enable context help** item in the list. To disable context help, click on **Help** on the menu bar and select **Enable context help** again. The check mark should now be gone.

To now get information on a term place the mouse over that term. The mouse should now change to a box with a question mark.

Click on the field name while the question mark is on it. A new window should appear with information about the particular term.

It is important to be aware that while it is part of the role of the data modeller to enter the information for the context help, that information is provided by the end user or some other domain expert. If there are gaps in the information or there is in fact no information available for any given term then speak the data modeller about adding information provided by you or your organization.
