# Supplementary material for: Information management for high content live cell imaging
Source: BMC Bioinformatics. 2009 Jul 21;10:226. doi: 10.1186/1471-2105-10-226 (PMC2723092; doi:10.1186/1471-2105-10-226)
Supplement: Additional file 5 — Pre-configured Pedro data capture tool. Pedro data capture tool configured to function with eXist XML database. [file 1471-2105-10-226-S5.zip › configuredpedro/doc/tutorials/user/EditingFiles.html]

Pedro User Tutorial - Lessons about Data Entry


## Pedro Tutorials

### User Tutorials

  
Pedro User Tutorial Overview  
Parts of a Pedro Window   
File Management  
File Editing  
Templates  
Importing Data  
Backup Files  
Viewing  
Searching  
Ontologies  
Context Help  
Exporting Files  
Alerts  
  
  

### Links

  
Main Tutorial Page  
Pedro Main Page  
Contact

## File Editing

  

### Learn how to ...

- copy and paste;
- create new subrecords;
- keep changes made to a subrecord;
- delete records;
- cancel record changes.

### Copying and Pasting

Copying and pasting can apply to either text for field values on a form or subrecords.

Copying and pasting text for field values is done by first highlighting the text you want to copy. Then click
on **Edit** on the menu and then select **Copy**. This puts a copy of the value in a buffer. Then to
paste, go to the field that you want to fill in. Click on the space for the value to make sure that the cursor
is in the right place. Then, click on **Edit** on the menu and select **Paste**. The value should now be
in your chosen field.

Copying and pasting a subrecord is done by first selecting the subrecord you wish to copy on the tree on the
left of the Pedro window. Click **Edit** on the menu and then select **Copy**. Then, on the tree on the
left hand side of the Pedro window, go to where everelse this subrecord would occur in the model. For example,
in the cancerPatientRecord model we may wish to copy the **Demograpchic** subrecord. Having copied it we
then go to another branch of the main tree and follow it down until we get to the same place where
**Demographic** is. Then click on **Edit** on the menu and select **Paste**. The contents of the
copied subrecord should now be in place.

The **Move Up** and **Move Down**
buttons at the bottom of the tree allow the user to re-order entries in the tree that occur in the same
level. From the figure below, **Demographics**, **Laboratory Results**, and **Treatments** are all on
the same level.
Selecting one of these and then clicking one of the **Move Up** or **Move Down** buttons will change
the order of these entries. It will not move entries up and down a branch of the tree - these buttons are
not for navigation. If there are entries within the item being moved, then these will be packaged with the
selected entry and moved with them so as not to distort the tree.

If you try to paste a subrecord in an inappropriate spot, Pedro will give you an error message.

### Creating New Subrecords

All fields that can have subrecords have two buttons next to them on the form: **New** and **Edit**. To
create a new subrecord from the form you are on, click the **New** button and this should then take you to
the subrecord of the form you were just on.

### Keeping Changes Made to a Subrecord

To keep the values on a form that you have filled in, click the **Keep** button at the bottom of the form.
Note that this only keeps the data on that record and does not save the entire session. Clicking the
**Done** button does the same thing as **Keep** except that it also takes you back to the parent record
of the one you were just on.

### Deleting Records

To delete a record, click on the record you wish to delete on the tree on the left of the Pedro window. At
the bottom of the form itself click the **Delete** button. This should remove the record from the tree
on the left and the right hand side of the Pedro wiindow should now display the parent of the record
just deleted. Be aware that if you delete a record that has subrecords of its own, then all the subrecords
will be delted as well.

### Cancelling Record Changes

If you make changes to a record and want to cancel them, click the **Cancel** button at the bottom of the
form. Note that if you save or keep your changes to the form, you cannot remove them by cancelling.
