# Supplementary material for: Information management for high content live cell imaging
Source: BMC Bioinformatics. 2009 Jul 21;10:226. doi: 10.1186/1471-2105-10-226 (PMC2723092; doi:10.1186/1471-2105-10-226)
Supplement: Additional file 5 — Pre-configured Pedro data capture tool. Pedro data capture tool configured to function with eXist XML database. [file 1471-2105-10-226-S5.zip › configuredpedro/doc/tutorials/user/Exporting.html]

Pedro User Tutorial - Lessons about Data Entry


## Pedro Tutorials

### User Tutorials

  
Pedro User Tutorial Overview  
Parts of a Pedro Window   
File Management  
File Editing  
Templates  
Importing Data  
Backup Files  
Viewing  
Searching  
Ontologies  
Context Help  
Exporting Files  
Alerts  
  
  

### Links

  
Main Tutorial Page  
Pedro Main Page  
Contact

## Exporting Files

  

### Learn how to ...

- export files to XML format.

Saving a file creates a PDZ format. However, Pedro has the ability to export files to XML format so that you can do other things with your data files.

To export a file click on **File** on the menu and select **Export to Final Submission Format...**. A dialogue window should now appear prompting you to save your file. As long as the **Files of Type:** field has the default value of **XML experiment files** then the file saved should automatically be in XML format.
