# Supplementary material for: Information management for high content live cell imaging
Source: BMC Bioinformatics. 2009 Jul 21;10:226. doi: 10.1186/1471-2105-10-226 (PMC2723092; doi:10.1186/1471-2105-10-226)
Supplement: Additional file 5 — Pre-configured Pedro data capture tool. Pedro data capture tool configured to function with eXist XML database. [file 1471-2105-10-226-S5.zip › configuredpedro/doc/tutorials/user/ImportingData.html]

Pedro User Tutorial - Lessons about Data Entry


## Pedro Tutorials

### User Tutorials

  
Pedro User Tutorial Overview  
Parts of a Pedro Window   
File Management  
File Editing  
Templates  
Importing Data  
Backup Files  
Viewing  
Searching  
Ontologies  
Context Help  
Exporting Files  
Alerts  
  
  

### Links

  
Main Tutorial Page  
Pedro Main Page  
Contact

## Importing Data

  

### Learn how to ...

- import data from a tab-delimited text file.

### Importing tab-delimited text files

Pedro allows you to import spreadsheet data in the form of a
tab-delimited text file. Most popular spreadsheet programs, such as Microsoft Excel, will allow you to
export data as a tab-delimited file.

Multiple lines in a tab-delimited file correspond to mulitple records on a Pedro form and you must make sure that that the record you are populating allows for multiple subrecords. If you try to import a file that has multiple lines into a record that does not support multiple subrecords you will get an error. Pedro will also validate the current record before you attempt to import data into it. To avoid Pedro detecting any errors on the form before you start importing, ensure that all required fields have been filled in and all filled in fields have the right kind of values, eg dates have been filled in correctly and fields expecting numbers have numbers.

To begin importing, make sue you have selected the parent record of the record you wish to populate in the tree. Click on **File** on the menu and select **Import Records** and then click on **Default Text Import...**. A dialouge window should now appear called Select Import Destination Field.

You should check on the left hand list labeled **List Fields** that the record you want to import to is listed. Note that the entries listed here are the children of the record you selected on the tree in the main Pedro window. Click on the entry you want to populate. In the right hand list labeled **Child Record Types** there will now be listed the type of subrecords that can be made within the particular record selected from the left. Click on the entry that is appropriate for you nd then click **OK**. A new window should appear called **Stripping Header Lines**. What this is actually doing is trying to determine where the tab-delimited file should start being read from. Click on the row that you want to be the start of the import. If you aren't sure then just click the top row and then click **OK**. A new window should appear called **Matching Columns to Record Fields**.

This window, as the name indicates, is where you match up the columns formed from the tab-deliimited file to the fields in the record you have selected. At the top of each column, right click and a list should appear. The entries in this list are the names of the fields within the record you are populating. Just select the name of the field you want populated with the data in the column. When you are done click the **Import** button. Importing is now complete for this record.

After the importing has been completed you will notice that the values of the imports now appear in the record you selected and also in the tree on the left of the Pedro window. It is important to remember that Pedro can currently only import tab-delimited text files and not files such as Microsoft Excel's \*.xls files.
