# Supplementary material for: Information management for high content live cell imaging
Source: BMC Bioinformatics. 2009 Jul 21;10:226. doi: 10.1186/1471-2105-10-226 (PMC2723092; doi:10.1186/1471-2105-10-226)
Supplement: Additional file 5 — Pre-configured Pedro data capture tool. Pedro data capture tool configured to function with eXist XML database. [file 1471-2105-10-226-S5.zip › configuredpedro/doc/tutorials/user/Management.html]

Pedro User Tutorial - Lessons about Data Entry


## Pedro Tutorials

### User Tutorials

  
Pedro User Tutorial Overview  
Parts of a Pedro Window   
File Management  
File Editing  
Templates  
Importing Data  
Backup Files  
Viewing  
Searching  
Ontologies  
Context Help  
Exporting Files  
Alerts  
  
  

### Links

  
Main Tutorial Page  
Pedro Main Page  
Contact

## File Management

  

### Learn how to ...

- open a file;
- create a file;
- save a file with a file name;
- save changes to a file;
- close a file;
- exit the program.

### Opening a File

To open a file in Pedro, on the menu bar click on File and the select Open... . A file chooser dialouge box should then appear. Select the file you wish to open. This file will have to be based on the model that you selected when initially starting Pedro. For example, if you select the cancerPatientRecord model initially then the file you select to open must have been created using this model. If the file you select does not open, make sure that you've go the right model to start.

### Creating a File

To create a new file, on the menu bar click File and select New. A new Pedro window should appear with the same model as was originally loaded. To open a new model you must re-run the programme and select the new model.

### Saving a File with a File Name

To save a file with a file name, on the menu bar click File and select Save As... . A file chooser dialouge box should then appear. Type a name for the file and select a directory where you want to keep the file.

### Saving Changes to a File

To save changes to a file, on the menu bar click File and select Save. This will save your changes with no change to the name of the file.

### Closing a File

To close a file, on the menu bar click File and select Close. This closes the current session of Pedro. If you have other sessions open then they will stay open.

### Exiting the Programme

To exit the Pedro application, on the menu bar click File and select Exit. This will end all sessions of Pedro. You will be prompted to save and changes on each session that you haven't alreday saved.
