# Supplementary material for: Information management for high content live cell imaging
Source: BMC Bioinformatics. 2009 Jul 21;10:226. doi: 10.1186/1471-2105-10-226 (PMC2723092; doi:10.1186/1471-2105-10-226)
Supplement: Additional file 5 — Pre-configured Pedro data capture tool. Pedro data capture tool configured to function with eXist XML database. [file 1471-2105-10-226-S5.zip › configuredpedro/doc/tutorials/user/Ontologies.html]

Pedro User Tutorial - Lessons about Data Entry


## Pedro Tutorials

### User Tutorials

  
Pedro User Tutorial Overview  
Parts of a Pedro Window   
File Management  
File Editing  
Templates  
Importing Data  
Backup Files  
Viewing  
Searching  
Ontologies  
Context Help  
Exporting Files  
Alerts  
  
  

### Links

  
Main Tutorial Page  
Pedro Main Page  
Contact

## Ontologies

  

### Learn how to ...

- get possible field values from ontologies.

There does not appear to be a set definition for the term ontology. In the context of Pedro, an
onotology is a controlled vocabulary that gives terms as they are meant to be presented (eg. spelt)
and the context (eg ordered) in which they are meant to be found. Ontologies can be used when you
are uncertain as to which terms are appropriate for a given field. This is dependent upon the
existance of an ontology which has the term you are looking for.

### Links to an ontology

Fields in a model that are linked to some kind of ontology are marked with an \*. Fields linked to ontologies may allow you to add terms not in the ontology if the field can be edited. However, if the field cannot be edited then you may only use terms from the onotology to fill the field in. The list of ontologies is accessed by right clicking on the field name. The list you see at this stage is the list of ontologies associated with the given field name. There may be more than one ontology linked to a particular term. There may also be a **Clear Values** entry in the list which allows you to clear the value selected. To access the list of ontology terms themselves, move the mouse over the ontology you want. A list of terms should now appear.

Ontologies can come from many sources but for the end user of Pedro, what is important is how the terms are selected for field entry. Depending on the number of terms in the ontology Pedro will display the options in slightly different ways. If there are only a few terms then the a list will be presented listing the terms alphabetically. If the number of terms is larger then Pedro then groups the temrs into sublists, again ordered alphabetically. If there are more than 40 terms then Pedro renders the terms alphabetically in a new window called a **Controlled Vocabulary Viewer** with the ability to search for a term in the list. Click the **Use Terms** button to enter the value in the field on the main Pedro window. **Close** closes **Controlled Vocabulary Viewer**.

If you would like to see more of this, please download Pedro Ontology Services Demo available on the Pedro download page.
