# Supplementary material for: Information management for high content live cell imaging
Source: BMC Bioinformatics. 2009 Jul 21;10:226. doi: 10.1186/1471-2105-10-226 (PMC2723092; doi:10.1186/1471-2105-10-226)
Supplement: Additional file 5 — Pre-configured Pedro data capture tool. Pedro data capture tool configured to function with eXist XML database. [file 1471-2105-10-226-S5.zip › configuredpedro/doc/tutorials/user/PedroParts.html]

Pedro User Tutorial - Lessons about Data Entry


## Pedro Tutorials

### User Tutorials

  
Pedro User Tutorial Overview  
Parts of a Pedro Window   
File Management  
File Editing  
Templates  
Importing Data  
Backup Files  
Viewing  
Searching  
Ontologies  
Context Help  
Exporting Files  
Alerts  
  
  

### Links

  
Main Tutorial Page  
Pedro Main Page  
Contact

## Parts of a Pedro Window

  

### Learn how to ...

- recognize the menus, record tree, and record form and what they do;
- recognize different kinds of data fields in the record form;
- work the control buttons in the lower right pat of the record form.

### The full screen

The Pedro GUI consists of three parts: the menu bar, the record tree, and the record form. Other
lessons will go into greater detail about each feature but for now let us get a high level look. The
main body of the application window is divided into two parts.
The left part of the window is the record tree that lists records and
subrecords in an experiment file. The right part shows a record form
for the currently selected record in the tree.

In the directory, double click on the run\_tutorial MS-DOS Batch File to start the tutorial example.

### The Menu Bar

The menu bar is found at the top left of the Pedro GUI.

Below is a summary of the various feature within each menu item.

|  |  |  |  |  |  |  |  |  |  |  |  |  |  |  |  |  |  |  |  |  |  |  |  |
| --- | --- | --- | --- | --- | --- | --- | --- | --- | --- | --- | --- | --- | --- | --- | --- | --- | --- | --- | --- | --- | --- | --- | --- |
| Feature | Description || New | causes a new blank Pedro window to appear. |
| Open... | opens an experiment file. |
| Save | saves any changes you've made to disk. |
| Save As... | saves the data you have to disk, in a file with a name you specify. |
| Close | closes the current file. |
| Import Records | allows you to import the rows of a tab-delimited text file as subrecords for the current file. |
| Favourites | allows you to see files that have been created with the current model. If no files have yet been created then this item will not appear on the drop down list. |
| Import from XML... | allows you to import from an XML file. |
| Export to Final Submission Format... | allows you to export the current file to different formats. |
| Templates | a submenu that allows you to save or load reusable "chunks" of an experiment file. This feature is useful in situations where curators are using the same set of conditions over multiple experiment files. |
| Exit | terminates the program. |

| Feature | Description |
| --- | --- |
| Copy | lets you copy one of two things to the clip board:  - highlighted text from a text field in the record form; - the currently selected record and all its subrecords in the record   tree.  Copied text can be pasted into applications such as Microsoft Word. |
| Paste | lets you paste copied text into the data field that shows the flashing cursor. It also lets you paste the contents of a copied record into the current record form. |

| Feature | Description |
| --- | --- |
| Show Errors | shows errors in the current file. An error will either mean that some edit field in the current record is incorrect, or that some record in the record tree is missing a required subrecord. |
| Show Dependencies... |  |
| Show Changes | shows changes you've made since the last time you saved your file to disk. Changed items in the record tree will appear either with a blue folder or a blue bullet. |
| Search... | lets you search through the subrecords. |
| Clear | clears all highlighting done by other features in the "View" menu. |

| Feature | Description |
| --- | --- |
| Alerts... | let's you validate your document by using additional checks made by other people |

| Feature | Description |
| --- | --- |
| File: | This window shows a list of the currently open files. Pressing a button in this menu will cause the window containing that file to appear in front of the others. |

| Feature | Description |
| --- | --- |
| About... | describes the team of people who made the software. |
| Schema Information... | tells you the name and version of the schema currently being used by the application. |
| Help... | takes you to the main help topic pages. |
| Enable context help | toggles context-sensitive help. When this feature is ticked, you can move the mouse cursor around and click for help on any place where a question mark cursor appears. |

### The Record Tree

The tree allows you to navigate through all records of your form. The **Move Up** and **Move Down**
buttons at the bottom of the tree allow the user to re-order entries in the tree that occur in the same
level. From the figure below, **Demographics**, **Laboratory Results**, and **Treatments** are all on the same level.
Selecting one of these and then clicking one of the **Move Up** or **Move Down** buttons will change
the order of these entries. It will not move entries up and down a branch of the tree - these buttons are
not for navigation. If there are entries within the item being moved, then these will be packaged with the
selected entry and moved with them so as not to distort the tree.

### The Record Form

The record form shows the data fields for the currently selected record and
a set of control buttons located in the bottom-right for saving,
deleting and cancelling operations.

### Form Fields

Required data fields appear in bold print while optional fields are in regular print. Fields can be
directly editable or modified only by
accessing subrecords or values linked to an ontology. A field that is has an associated ontology i
s marked with a \* character before it. All list fields have a **New** and **Edit** buttons that
correspond to creating a subrecord and editing a subrecord respectively. Fields with enumerated
values appear either as a pre-set list or as radio buttons. These kinds of fields do not have **New**
or **Edit** buttons.

The appearance of list fields is effected by two factors:

- they can hold multiple subrecords or only one
- they can hold multiple types of subrecords, or only one

List fields that can contain multiple subrecord types appear with a
**Show:** label and a combination field with choices for subrecord
type. The combination field value is used in two ways:

- filters the list display to show only subrecords of the selected type
- sets the type of subrecord that is created when you create a new
  subrecord.

Fields can also contain web page adresses as shown below. The URL can be typed in manually into the field
or it can be selected from a list by clicking the **Browse...** button. Clicking the **View** button
brings the specified web page up. You will not be able to click directly on the URL listed in the field.

### Control Buttons

A group of four buttons always appears in the bottom-right part
of the record form. If they appear to be greyed out then this means that this particular feature is
unavailable until some change is made to the form.

| Feature | Description |
| --- | --- |
| Done | saves the changes you've made to the currently displayed record, stores them in memory, and then automatically takes you back to the parent record of the record you were just on. |
| Keep | saves the changes you've made to the currently displayed record and stores them in memory. |
| Cancel | restores the last saved values of the data fields for the current record. |
| Delete | deletes the record from the file. |
