# Supplementary material for: Information management for high content live cell imaging
Source: BMC Bioinformatics. 2009 Jul 21;10:226. doi: 10.1186/1471-2105-10-226 (PMC2723092; doi:10.1186/1471-2105-10-226)
Supplement: Additional file 5 — Pre-configured Pedro data capture tool. Pedro data capture tool configured to function with eXist XML database. [file 1471-2105-10-226-S5.zip › configuredpedro/doc/tutorials/user/Searching.html]

Pedro User Tutorial - Lessons about Data Entry


## Pedro Tutorials

### User Tutorials

  
Pedro User Tutorial Overview  
Parts of a Pedro Window   
File Management  
File Editing  
Templates  
Importing Data  
Backup Files  
Viewing  
Searching  
Ontologies  
Context Help  
Exporting Files  
Alerts  
  
  

### Links

  
Main Tutorial Page  
Pedro Main Page  
Contact

## Searching

  

### Learn how to ...

- search for record names in the record tree.

There are two kinds of searching supported by Pedro: simple and advanced.

### Simple Searching

To do a simple search for a field value in the data set, click on **View** on the menu and then select **Search...**. A new window should appear called **Search Dialog**. Enter the value you are searching for in the field labelled **Find:**. It can be the whole value or a partial string. Then click the **Search** button.

In the field below it called **Search Results** there will appear a listing of the records that contain the value you are searching for. If you type in more terms to find, their search results will be added to the list. If you wish to only have one set of results listed then click the **Clear** button and this will clear both the fields in the window. If you do not do this then every time you search during the current Pedro session, the results of previous searches will appear in the **Search Results** field.

### Advanced Searching

The second kind of searching is advanced. This allows you to be more specific in narrowing your searching. To begin the advanced search start as you would a simple search to get the **Search Dialog** window up. On this window, then click the tab labelled **Advanced Search**. Right below the tabs is a field labelled **Select:**. In this pull down list are the names of all the records that exist in the model. If you select a record that has no subrecords then the button labelled **Add Edit Field Criterion...** will be highlighted. If you select a record that has subrecords then the button labelled **Add List Field Criterion...** will be highlighted. It is with either of these buttons that you can narrow your search.

Whether you are looking at a field or a record, there will be a pull down list of values that are either field names in the record you are searching or subrecord names in the record you are searching. You can then perform a variety of searches based on whether a value matches or does not match the value you enter or whether the value is greater or less than what you are searching for.

In all cases, the results of your search will be shown in the bottom field labelled **Search Results**. Clearing your results is done the same way as in simple searching.
