# Supplementary material for: Information management for high content live cell imaging
Source: BMC Bioinformatics. 2009 Jul 21;10:226. doi: 10.1186/1471-2105-10-226 (PMC2723092; doi:10.1186/1471-2105-10-226)
Supplement: Additional file 5 — Pre-configured Pedro data capture tool. Pedro data capture tool configured to function with eXist XML database. [file 1471-2105-10-226-S5.zip › configuredpedro/doc/tutorials/user/StartingTutorial.html]

Pedro User Tutorial - Lessons about Data Entry


## Pedro Tutorials

### User Tutorials

  
Pedro User Tutorial Overview  
Parts of a Pedro Window   
File Management  
File Editing  
Templates  
Importing Data  
Backup Files  
Viewing  
Searching  
Ontologies  
Context Help  
Exporting Files  
Alerts  
  
  

### Links

  
Main Tutorial Page  
Pedro Main Page  
Contact

## User Tutorial - Learning About Pedro and Data Entry

  

Pedro is an application that produces data entry forms for a data model that is specified in a particular style of XML Schema. If you're only going to do data entry with the tool, don't worry about schemas because you should never have to see one. The sections below are designed for people who will only be doing data entry with the tool.

Pedro was originally developed for the proteomics community and we
ship their data model for those scientists to use. To Pedro however, the data model
could be about proteomics, botany,
chemistry, or about your favourite bird calls or comic books!
This tutorial however uses
a mock data model for patient medical records.

The model that is used for this tutorial is called cancerPatientRecord. No specific files have been created for it. If you want to practice on some of the aspects of the tutorial then start Pedro and use this model. All the screen shots have been taken from various uses of this model so it might be helpful to pick this model.

Why talk about medical records if the application was originally targetting another field? We intentionally used a different tutorial model for the following reasons:

- we don't want to make your experience learning about the tool more complicated by forcing you to learn about a complicated data model;
- using a live data model for a tutorial is a bad idea, because if the data model changes, the text and screen shots have to change also;
- we want to demonstrate that this is a generic tool that can handle data models from completely different fields.

The sections listed in the menu on the left summarise the various aspects of the Pedro application. While you are free to look at the in any order, it is suggested that you familiarise yourself with the Parts of a Pedro Window first as the content of this will be referred to in other sections. Words that appear in bold (eg **Edit**) refer to elements that appear on the application.
