# Supplementary material for: Information management for high content live cell imaging
Source: BMC Bioinformatics. 2009 Jul 21;10:226. doi: 10.1186/1471-2105-10-226 (PMC2723092; doi:10.1186/1471-2105-10-226)
Supplement: Additional file 5 — Pre-configured Pedro data capture tool. Pedro data capture tool configured to function with eXist XML database. [file 1471-2105-10-226-S5.zip › configuredpedro/doc/tutorials/user/Templates.html]

Pedro User Tutorial - Lessons about Data Entry


## Pedro Tutorials

### User Tutorials

  
Pedro User Tutorial Overview  
Parts of a Pedro Window   
File Management  
File Editing  
Templates  
Importing Data  
Backup Files  
Viewing  
Searching  
Ontologies  
Context Help  
Exporting Files  
Alerts  
  
  

### Links

  
Main Tutorial Page  
Pedro Main Page  
Contact

## Templates

  

### Learn how to ...

- save a template;
- load a template.

Copying and pasting records between files is useful in situations
where part of one file is similar to part of another. Templates
represent records that don't change and that can imported into multiple
experiment files.

### Saving Templates

To save a template, click on **File** on the menu bar and then move the mouse over **Templates**. You will given a choice to click either **Save Template** or **Open Template**. For this, click **Save Template**. A dialouge should appear prompting you to save a file.

### Opening Templates

To open a template, you first need to go to the record on the tree that you wish to poulate with the template. Then click on **File** on the menu and select **Templates** then **Open**. the form should now look populated with the values saved in the template. It is important to remember that the values in a loaded template will overwrite whatever was in the fields initially. If you try to open a template in an inappropriate place Pedro will give you an error.
