# Supplementary material for: Information management for high content live cell imaging
Source: BMC Bioinformatics. 2009 Jul 21;10:226. doi: 10.1186/1471-2105-10-226 (PMC2723092; doi:10.1186/1471-2105-10-226)
Supplement: Additional file 5 — Pre-configured Pedro data capture tool. Pedro data capture tool configured to function with eXist XML database. [file 1471-2105-10-226-S5.zip › configuredpedro/doc/tutorials/user/UserTutorial.html]

Pedro User Tutorial - Lessons about Data Entry


## Pedro Tutorials

### User Tutorials

  
Starting the Pedro Tutorial  
Parts of a Pedro Window   
File Management  
File Editing  
Templates  
Importing Data  
Backup Files  
Viewing  
Searching  
Ontologies  
Context Help  
Exporting Files  
  
  

### Links

  
User Tutorial Page  
Main Tutorial Page  
Pedro Main Page

## User Tutorial - Learning about Data Entry

  


Pedro is an application that produces data entry forms for a data
model that is specified in a particular style of XML Schema. If
you're only going to do data entry with the tool, don't worry about schemas
because you should never have to see one. The lessons below are designed for people who will only be doing data
entry with the tool.

Pedro was originally developed for the proteomics community and we
ship their data model for those scientists to use. To Pedro however, the data model
could be about proteomics, botany,
chemistry, or about your favourite bird calls or comic books!
This tutorial however uses
a mock data model for patient medical records. Why talk about medical records if
the application was originally targetting
another field? We
intentionally used a different tutorial model for the following reasons:

- we don't want to make your experience learning about the tool more
  complicated by forcing you to learn about a complicated data model
- using a live data model for a tutorial is a bad idea, because if
  the data model changes, the text and screen shots have to
  change also.
- we want to demonstrate that this is a generic tool that can handle data
  models from completely different fields.
