# Supplementary material for: Information management for high content live cell imaging
Source: BMC Bioinformatics. 2009 Jul 21;10:226. doi: 10.1186/1471-2105-10-226 (PMC2723092; doi:10.1186/1471-2105-10-226)
Supplement: Additional file 5 — Pre-configured Pedro data capture tool. Pedro data capture tool configured to function with eXist XML database. [file 1471-2105-10-226-S5.zip › configuredpedro/doc/tutorials/user/Viewing.html]

Pedro User Tutorial - Lessons about Data Entry


## Pedro Tutorials

### User Tutorials

  
Pedro User Tutorial Overview  
Parts of a Pedro Window   
File Management  
File Editing  
Templates  
Importing Data  
Backup Files  
Viewing  
Searching  
Ontologies  
Context Help  
Exporting Files  
Alerts  
  
  

### Links

  
Main Tutorial Page  
Pedro Main Page  
Contact

## Viewing

  

### Learn how to ...

- recognize changes in the record tree when viewing;
- use different kinds of views.

### The kinds of viewing

Pedro allows users to view three different aspects of the model being used. These are errors that
have occured in the model, dependencies within the model, and changes made to the model. In general, the
features for viewing apply to the entire tree/form irrespective of which tree entry/record you are
on. Viewing is done by means of selecting an entry on the record tree which then becomes the record
shown in the form side of the GUI.

### Viewing Errors

A record will have an error if:

- it is the currently displayed record, and it has illegal edit
  field values. These values will appear red in the record form.
- it lacks a required subrecord in one or more of its list fields.

Pressing **Keep** will display any errors on that particular form.

To view errors throughout the session, click on **View** on the menu and select **View Errors**. A new window will appear
called **Alerts Detected**. This will display not only errors that occur on the particular record you're on but also errors that
have occurred throughout the rest of the session.

### Viewing Dependencies

You may be using Pedro with a schema that supports notions of referencing records in some other part of the document. For more
information on this, please consult your data modeller. In these cases, you may want to know which records reference the currently
selected selected one. This may be important in case you want to assess the impact of deleting the referenced record.

Selecting **Show Dependencies** will highlight all the records that reference the currently selected one. You can unhighlight the results
by pressing **Clear** in the **View** menu.

### Viewing Changes

**Show Changes** will show you which parts of your file have been
modified since the last time you saved your file. Note that this does not apply to the last time you pressed **Keep** for a
particular record.

A record will be flagged as a changed record if:

- its edit fields have changed value
- it is a new subrecord
- a subrecord has been added or removed.

To view changes, click on **View** on the menu and select **View Changes**. You'll observe that in the record tree of a file, the
item is accompanied by
either a yellow folder or a gray bullet. Show changes will cause these icons to appear blue if that record has been changed.

Changes to the entire data set can be viewed from any record and will still show all changes that have occurred throughout the data set. You
can clear the tree of these markups by clicking on **View** on the menu bar and then selecting **Clear**. The blue highlights will
then disappear.
