# Supplementary material for: Information management for high content live cell imaging
Source: BMC Bioinformatics. 2009 Jul 21;10:226. doi: 10.1186/1471-2105-10-226 (PMC2723092; doi:10.1186/1471-2105-10-226)
Supplement: Additional file 5 — Pre-configured Pedro data capture tool. Pedro data capture tool configured to function with eXist XML database. [file 1471-2105-10-226-S5.zip › configuredpedro/models/Cell_Characteristics/doc/atomic_action_cv.html]

**atomic\_action\_cv***Procedures used during treatment*
  
*An atomic action is a single step process on the biomaterial, e.g change\_light,**remove add*

---

Model The MGED Ontology V "1.1.6"
  
  
class Action
  
class AtomicAction  


---

add  
change\_light  
change\_temperature  
remove  
set\_temperature
