# Supplementary material for: Information management for high content live cell imaging
Source: BMC Bioinformatics. 2009 Jul 21;10:226. doi: 10.1186/1471-2105-10-226 (PMC2723092; doi:10.1186/1471-2105-10-226)
Supplement: Additional file 5 — Pre-configured Pedro data capture tool. Pedro data capture tool configured to function with eXist XML database. [file 1471-2105-10-226-S5.zip › configuredpedro/models/Cell_Characteristics/doc/Characteristics_cv.html]

**Characteristics\_cv**
  
  
*Properties of the biomaterial before treated in any manner
  
for the purposes of the experiment.
  
Such as strain or line.*


---

Model The MGED Ontology V "1.1.6"
  
  
class BioMaterialCharacteristic
  
class BioMaterialCharacteristicCategory
  


---

  
age
  
The time period elapsed since an identifiable point in the life cycle of
an organism. If a developmental stage is specified, the identifiable point
would be the beginning of that stage. Otherwise the identifiable point must be
specified such as planting.

bedding
  
Refers to the bedding material present in an animals housing.

biomaterial\_provider
  
The resource (e.g, company, hospital, geographical location) used to obtain or
purchase the biomaterial.

biometrics
  
Physical properties of the BioMaterial e. g. mass or height

cell\_line

cell\_type
  
CellType, the type of cell used in the experiment if non mixed, if mixed
the TargetedCellType should be used, example of instances, epithelial, glial
etc.

chromosomal\_aberration\_classification
  
An irregularity in the number or structure of chromosomes, usually in the form
of a gain (duplication), loss (deletion), exchange (translocation), or
alteration in sequence (inversion) of genetic material. Excludes simple changes
in sequence such as mutations, and is usually detectable by cytogenetic and
microscopic techniques such as FISH.

clinical\_information
  
clinical\_treatment
  
The current clinical treatment(s) of the patient from which the biosource is
derived.

developmental\_stage
  
The developmental stage of the organism's life cycle during which the
biomaterial was extracted.

disease\_staging
  
The stage or progression of a disease in an organism. Includes
pathological staging of cancers and other disease progression.

disease\_state
  
The name of the pathology diagnosed in the organism from which the
biomaterial was
  
derived.
  
The disease state is normal if no disease has been diagnosed.

environmental\_history
  
some aspect of the organism's environmental history, such as exposure to
teratogen, radiation, climate etc.

genetic\_modification
  
The genetic modification introduced into the organism from which the
biomaterial was derived. Examples of genetic variation include specification of
a transgene or the gene knocked-out.

histology
  
Microscopic morphology of tissues.

individual
  
Identifier or name of the individual organism from which the biomaterial was
derived.

individual\_genetic\_characteristic
  
The genotype of the individual organism from which the biomaterial was derived.
Individual genetic characteristics include polymorphisms, disease alleles, and
haplotypes.

organism
  
The genus and species (and subspecies) of the organism from which the
biomaterial is derived from.

organism\_part
  
The part of the organism's anatomy from which the biomaterial was derived,
excludes cells. E.g. tissue, organ, system, or body location (arm).

organism\_status
  
The stage premortem or postmortem at which the sample was processed for
extraction of biomaterials.

phenotype
  
The observable form taken by some character (or group of characters) in an
individual or an organism, excluding pathology and diesease. The detectable
outward manifestations of a specific genotype.

sex
  
F+
  
F-
  
Hfr
  
female
  
hermaphrodite
  
male
  
mating\_type\_a
  
mating\_type\_alpha
  
mixed\_sex
  
unknown\_sex

strain\_or\_line
  
Animals or plants that have a single ancestral breeding pair or parent as a
result of brother x sister or parent x offspring matings.

targeted\_cell\_type
  
The target cell type is the cell of primary interest. The biomaterial may be
derived from a mixed population of cells although only one cell type is of
interest.

tumor\_grading
  
A descriptor used in cancer biology to describe abnormalities of tumor cells.
E.g. an instance from NCI Thesaurus.
