# Supplementary material for: Information management for high content live cell imaging
Source: BMC Bioinformatics. 2009 Jul 21;10:226. doi: 10.1186/1471-2105-10-226 (PMC2723092; doi:10.1186/1471-2105-10-226)
Supplement: Additional file 5 — Pre-configured Pedro data capture tool. Pedro data capture tool configured to function with eXist XML database. [file 1471-2105-10-226-S5.zip › configuredpedro/models/Cell_Characteristics/doc/complex_action_cv.html]

**complex\_action\_cv**  
  
*Procedures used during treatment*
  
*Complex actions are composed of multiple steps (as opposed to AtomicAction)
e.g. , genetic\_modification.* 
  
  


---

Model The MGED Ontology V "1.1.6"  
  
class Action
  
class ComplexAction
  


---

change\_biomaterial\_characteristics  
genetic\_modification  
incubate  
infect  
irradiate  
split  
starvation  
transfect  
wash
