# Supplementary material for: Information management for high content live cell imaging
Source: BMC Bioinformatics. 2009 Jul 21;10:226. doi: 10.1186/1471-2105-10-226 (PMC2723092; doi:10.1186/1471-2105-10-226)
Supplement: Additional file 5 — Pre-configured Pedro data capture tool. Pedro data capture tool configured to function with eXist XML database. [file 1471-2105-10-226-S5.zip › configuredpedro/models/Cell_Characteristics/doc/Compound.html]

**Compound**

*A drug, solvent, chemical, etc., with a property that can be measured such as
concentration.*

---

Model from MAGE OM.  
  
Package BioMaterial
  
Class Compound
  


---

name

concentration  
Concentration of Compound

isSolvent
  
A Compound may be a special case Solvent

MerckIndex
  
The Merck Index of this Compound

ExternalLIMS  
Reference to an entry in an external LIMS data source
