# Supplementary material for: Information management for high content live cell imaging
Source: BMC Bioinformatics. 2009 Jul 21;10:226. doi: 10.1186/1471-2105-10-226 (PMC2723092; doi:10.1186/1471-2105-10-226)
Supplement: Additional file 5 — Pre-configured Pedro data capture tool. Pedro data capture tool configured to function with eXist XML database. [file 1471-2105-10-226-S5.zip › configuredpedro/models/Cell_Characteristics/doc/material_type_cv.html]

**material\_type\_cv**
  
  
*Controlled terms for the state of the BioMaterial.  
Examples are population of an organism, organism, organism part, cell, etc.*

---

Model The MGED Ontology V "1.1.6"  
  
class MaterialType

---

DNA
  
Deoxyribonucleic acid.

cell
  
One or more dissociated cell(s) possibly heterogeneous, excluding single cell
organisms

cell\_lysate
  
cell lysate, a collection of cells whose membranes have been disrupted

cytoplasmic\_RNA
  
RNA obtained from the cytoplasm.

genomic\_DNA
  
high molecular weight DNA

molecular\_mixture
  
an unspecified collection of heterogeneous molecules, e.g. lipids, carbohydrate
, nucleic acids and proteins

nuclear\_RNA
  
RNA obtained from the nucleus.

organellar\_DNA
  
DNA from organelles such as the mitochondria or chloroplast.

organellar\_RNA
  
RNA obtained from an organelle, e.g., mitochondrion, ER, or chloroplast,
excluding the nucleus.

organism\_part
  
The part of the organism's anatomy from which the biomaterial was derived,
excludes cells. E.g. tissue, organ, system, or body location (arm).

polyA\_RNA
  
RNA which has been obtained by selection for polyA tracts. Exact
synonym:polyA+\_RNA Non-exact synonym: mRNA

protein
  
Polymer of amino acids.

synthetic\_DNA
  
DNA which is generated by chemical or enzymatic (non cellular) means. non-exact
synonym: cDNA

synthetic\_RNA
  
RNA which is generated by chemical or enzymatic (non cellular) means. E.g. T7
promotor generated RNA. Non-exact synonym: cRNA, complementary RNA aRNA,
amplified RNA

total\_RNA
  
Total cellular and nuclear RNA.

virus
  
one or more DNA or RNA based non cellular infective agent, including
bacteriophage

whole\_organism
  
one or more of any unicellular or multicellular pro or eukaryote, including
archaebacteria
