# Supplementary material for: Information management for high content live cell imaging
Source: BMC Bioinformatics. 2009 Jul 21;10:226. doi: 10.1186/1471-2105-10-226 (PMC2723092; doi:10.1186/1471-2105-10-226)
Supplement: Additional file 10 — Raw counts. Counts of True Positives, False Positives and False Negatives for the training and test data. [file 1471-2105-10-226-S10.doc]

| **Data Set** | **TP** | **FP** | **FN** |
| --- | --- | --- | --- |
| T1 | 7 | 5 | 0 |
| T2 | 16 | 3 | 2 |
| T3 | 13 | 4 | 1 |
| T4 | 34 | 1 | 2 |
| T5 | 11 | 8 | 0 |
| T6 | 41 | 3 | 5 |
| T7 | 65 | 11 | 4 |

Supplemental Table: Training data, raw results.

| **Data Set** | **TP** | **FP** | **FN** |
| --- | --- | --- | --- |
| 1 | 44 | 8 | 11 |
| 2 | 63 | 7 | 11 |
| 3 | 47 | 6 | 9 |
| 4 | 16 | 39 | 6 |
| 5 | 10 | 15 | 3 |
| 6 | 7 | 34 | 9 |
| 7 | 14 | 12 | 0 |
| 8 | 159 | 23 | 24 |

Supplemental Table: Test data, raw results.
