# Supplementary material for: Information management for high content live cell imaging
Source: BMC Bioinformatics. 2009 Jul 21;10:226. doi: 10.1186/1471-2105-10-226 (PMC2723092; doi:10.1186/1471-2105-10-226)
Supplement: Additional file 5 — Pre-configured Pedro data capture tool. Pedro data capture tool configured to function with eXist XML database. [file 1471-2105-10-226-S5.zip › configuredpedro/models/People/doc/Template.html]

## Data Model 2.3

Finlay Lab plant field studies data model
created January 2, 2003

### Description

### Changes from Model 2.2


### Record Fields

|  |  |  |  |  |  |
| --- | --- | --- | --- | --- | --- |
| **Field** | **Required** | **Type** | **Restrictions** | **Description** | **Example** |
|  |  |  |  |  |  |
|  |  |  |  |  |  |
|  |  |  |  |  |  |
|  |  |  |  |  |  |
|  |  |  |  |  |  |
|  |  |  |  |  |  |

### Field Notes
