# Supplementary material for: Information management for high content live cell imaging
Source: BMC Bioinformatics. 2009 Jul 21;10:226. doi: 10.1186/1471-2105-10-226 (PMC2723092; doi:10.1186/1471-2105-10-226)
Supplement: Additional file 5 — Pre-configured Pedro data capture tool. Pedro data capture tool configured to function with eXist XML database. [file 1471-2105-10-226-S5.zip › configuredpedro/ReleaseNotes-v1_9_1.html]

Pedro v1.9.1 Release Notes


# Pedro v1.9.1 Release Notes

## Release Date: November 25, 2005

We are pleased to announce the release of Pedro v1.9.1. The highlights
of this release are described below.- Contact Us
- Tutorials
- Enhancements
- Bug Fixes


---

## Contact Us

The following table of contacts shows who you can contact about the
project. If you encounter a bug in the software, please report it
using 
SourceForge

|  |  |  |
| --- | --- | --- |
| **Role** | **Name** | **Contact** |
| - Feature Development - Enhancements | Kevin Garwood | garwood@cs.man.ac.uk |
| - Documentation - Schemas - End-User Training - Plugin Development | Chris Garwood | cgarwood@cs.man.ac.uk |

---

## Tutorials

Please refer to the new tutorials

## Enhancements

### Context-help web pages rewritten

Since the first release, users have been able to use a
context-sensitive help feature to get information about various
buttons and controls on the screen. These web pages have been
rewritten to the standard of the tutorials.

### "Move Up" and "Move Down" buttons added to the record tree.

The Pedro display is divided into left and right parts of the screen.
On the left part is a tree of folders that represents the records in
the current data file. As each new record is created, it gets added to the
tree. In past releases, the records added to the tree appeared in the
order they were created. The "Move Up" and "Move Down" buttons now
allow users to reorder the subrecords that would appear in the tree.

### A "Done" button has been added to the main record form.

In past releases, Pedro had buttons "Keep", "Cancel" and "Delete".
Now there is a "Done" button, which does the same operation as "Keep",
but also takes you up to the the parent record. For example, suppose
that an Experiment record has a subrecord called "Sample". You've
finished editing something in "Sample" and you press "Done". Pedro
will save your changes to memory, then switch the currently displayed
record to "Experiment".

### Search facilities have improved

Pedro supports two kinds of search features. In the simple search feature, you type in a
phrase and the results return any record that contains that phrase.
In the advanced search feature, you can create a set of matching criteria which must all
be satisfied for a result to be returned.

In previous releases, the advanced search comparison operations
treated all the criteria values as letters. Now the operations will
interpret the values as integers, doubles, floats or strings,
depending on the data type of the data field you choose to make part
of the matching criteria. The comparison operators that are displayed
are appropriate for the data type of the search field. For example, a
field like "age" would be associated with "=", "!=", ">", ">=", "<", "<="
operators whereas "first\_name" would be associated with "=", "!=",
"contains", "not contains" and "matches expression". This last
"matches expression" operator allows you to specify search criteria
values that are regular expressions.

---

## Bug Fixes

### Source Forge Bug #1110633: Plugin Management Bug, Source Forge Bug #1123830: Plugin to populate root node, #1120826: Plugins button doesn't appear

Pedro has a facility that finds all plugins that can operate on the
type of currently selected record. If there are any plugins that
work, then a "Plugins" button appears. There were bugs seeing
this plugin but those have been fixed.

### Source Forge Bug #1283989: Pedro writes .pdr and .meta files out to working directory

This would have only been noticed by people who were reading Pedro
files from a read only location. When the program reads a .pdz file,
it temporarily does a dump of the zip file contents, then deletes it.
In previous releases, these files were being dumped to the working
directory. However, the program may not always have write-access to
these locations. The problem has been fixed by having Pedro dump
temporary files to the operating system's default temp directory.
